# Modeling Tenascin-C-rich metastatic tumor niches in engineered hydrogel biomaterials

**DOI:** 10.1016/j.actbio.2026.07.054

**Published:** 2026-07-30

**Authors:** Aakanksha Jha, Elizabeth R. Lawlor, Cole A. DeForest

**Affiliations:** a Department of Chemical Engineering, University of Washington, Seattle, WA, 98105, USA; b Ben Towne Center for Childhood Cancer and Blood Disorders Research, Seattle Children's Research Institute, Seattle, WA, USA; c Human Biology Division, Fred Hutchinson Cancer Center, Seattle, WA, USA; d Department of Pediatrics, University of Washington, Seattle, WA, USA; e Department of Bioengineering, University of Washington, Seattle, WA, 98105, USA; f Institute of Stem Cell & Regenerative Medicine, University of Washington, Seattle, WA, 98105, USA; g Department of Chemistry, University of Washington, Seattle, WA, 98105, USA; h Molecular Engineering & Sciences Institute, University of Washington, Seattle, WA, 98105, USA; i Institute for Protein Design, University of Washington, Seattle, WA, 98105, USA

**Keywords:** Hydrogels, Sarcoma, Tumor microenvironment, Extracellular matrix, Tenascin-C

## Abstract

The extracellular matrix (ECM) plays a pivotal role in shaping tumor behavior by providing biochemical and biophysical cues to cancer cells. Traditional 2D culture systems fail to recapitulate this complexity, and *in vivo* systems do not readily allow for interrogation of how individual ECM components influence tumor cell behavior. Here, we introduce a fully synthetic, tunable three-dimensional (3D) hydrogel that mimics a soft tissue tumor microenvironment (TME) to enable mechanistic studies of ECM–tumor interactions. The hydrogel features a proteolytically degradable poly(ethylene glycol) base network functionalized with integrin-binding peptides derived from ECM components collagen I and fibronectin. To model metastatic ECM and further tune the hydrogel, we incorporated a tenascin-C (TNC)-derived peptide. To study the impact of these tunable ECM parameters on cancer cell behavior, we encapsulated Ewing sarcoma (EwS) cells within the hydrogels. EwS is an aggressive bone and soft tissue tumor that commonly metastasizes to lung. Our studies demonstrated matrix-dependent growth and phenotypic variation of EwS cells. Specifically, the TNC peptide drove divergent tumor behaviors and induced cell state changes consistent with alterations induced by the native TNC protein. To facilitate downstream functional assays, the hydrogel incorporates sortase-degradable crosslinkers that enable non-perturbative recovery of encapsulated cells. This platform provides a reductionist and reproducible model for studying the ECM’s role in cancer cell biology while addressing long-standing challenges in polymeric hydrogel degradation and cell retrieval. Collectively, this work establishes a biomaterials-based framework to dissect EwS tumor–ECM interactions in a controllable 3D microenvironment.

**Statement of significance:**

Engineered biomaterials to model Ewing sarcoma (EwS) have been previously employed to study metastasis to the bone. While important efforts, platforms to study EwS metastasis in lung – the most common site of metastasis – have not been developed. Here, we introduce a user-programmable biomaterial designed to mimic the environment of soft tissue metastases. The platform features several attributes: 1) it is mechanically matched to lung tissue; 2) it is readily functionalized with extracellular matrix protein-derived peptides (e.g., Tenascin-C); 3) encapsulated cells can be retrieved in a “biologically invisible” manner via sortase-mediated gel degradation, enabling expanded downstream analysis. This platform provides a powerful, reproducible tool to dissect tumor behavior and identify new targets for cancer therapy.

## 1. Introduction

Cancers are complex, multi-dimensional ecosystems whereby overall phenotype is dictated by the 3D tumor microenvironment (TME) [1-3]. The solid TME is composed of both cellular (e.g., stromal and immune cells) and non-cellular components (e.g., extracellular matrix, ECM) [4]. Serving as a scaffold for cell-matrix interactions, the ECM is a dynamically complex microenvironment that supports cell adhesion, migration, proliferation, and differentiation, mediated through a variety of proteins including collagens (Col), fibronectin (FN), elastin, and laminin [5,6]. During physiological processes, cells constantly remodel their ECM, simultaneously producing and degrading ECM components. However, during tumor development, the ECM undergoes fundamental alterations that heavily influence tumor progression, invasion, metastasis, and treatment response [7,8]. Though specific ECM composition differs among different types of solid tumors, FN and Col are universally detected in the TME of solid tumors [4]. Current models do not readily permit the experimental tunability necessary to fully understand the role of the ECM in cancer progression [9].

Hydrogel biomaterials have proven invaluable tools towards better understanding cancer phenotype and progression [10,11]. These materials can be readily designed to mimic critical aspects of the native TME (e.g., dimensionality, viscoelasticity, biochemical composition), providing a more *in vivo*-like platform to help shed light on 3D signaling, proliferation, and chemoresistance [12-19]. Though useful, many biomaterial formulation strategies irreversibly entrap cells within non-degradable matrices making them inaccessible to downstream analysis. Recent efforts have demonstrated use of the *S. aureus* transpeptidase Sortase A (SrtA) method to recover previously encapsulated cells from hydrogels containing the enzyme’s substrate in its network backbone in a “biologically invisible” manner [20-23]. Incorporating TME-relevant peptides into sortase-degradable gels, we sought here to establish a reductionist platform to study soft tissue-metastasizing cancers.

Lung metastasis is a major cause of death for patients with solid tumors, including both carcinomas that affect adults and non-epithelial tumors such as sarcomas that present more commonly in young patients [24-27]. Ewing sarcoma (EwS) is a rare bone and soft tissue tumor that peaks in children and adolescents. Patients with EwS presenting with distant metastases have a very poor prognosis [28,29]. The most common sites of EwS metastasis are lung, bone marrow, and bone [30]. Previous work has shown that the tumor’s ECM as well as tumor:TME crosstalk play a major role in EwS tumor progression [31,32]. Despite the apparent critical role of the ECM in governing tumor establishment and disease progression, the specific ECM cues determining EwS fate remain poorly understood. FN, Col, and the glycoprotein Tenascin-C (TNC) are highly prevalent in sarcoma TMEs with TNC being heterogeneously present [33-37]. TNC has been implicated in driving EwS metastasis [31], altering EwS phenotype in TNC-rich versus -poor microenvironments. It is therefore pertinent to dissect TNC’s role in EwS progression. Despite TNC’s crucial role, the contributions of specific regions of the TNC protein in dictating EwS behavior are yet to be fully deciphered. To better understand TNC’s impact on EwS phenotype and growth, we propose to use reductionist soft tissue-mimicking models using peptides that enable elucidation of specific roles of whole proteins on cell behavior. Soft tissue mimicking *ex vivo* platforms to study the role of these ECM components in EwS are lacking.

In this work, we sought to develop a tunable 3D hydrogel model that would permit mechanistic dissection of the impact of specific ECM ligands, specifically TNC, on EwS biology. Utilizing FN-, Col-, and TNC-derived peptides, we deconstruct the complex native ECM into its essential functional units while facilitating scalability, tunability, and analyses that are not feasible *in vivo*. Fabricated using poly(ethylene glycol) (PEG, a biologically inert synthetic polymer), ECM-derived peptides, and a peptide crosslinker that is cleavable both by cell-secreted matrix metalloproteinases and exogenously administered SrtA, these hydrogels can be tuned to promote cell adhesion and matrix remodeling with high rigor and reproducibility. Our studies demonstrate excellent sarcoma cell survival and growth with pronounced biological changes when TNC is included in the hydrogel formulation, as well as the ability to recover cells from gels for downstream analysis. To the best of our knowledge, this is the first soft tissue-mimicking hydrogel designed to study how different ECM components affect EwS biology, opening new doors for *ex vivo* study of the metastatic sarcoma niche.

## 2. Materials and methods

### 2.1. Cell culture and maintenance

CHLA10 EwS cell line was cultured in Iscove’s Modified Dulbecco’s Medium (IMDM) (Corning, Corning, NY) supplemented with 20% fetal bovine serum (FBS) (Atlanta Biologicals, Lawrenceville, GA), 1% insulin transferring selenium (ITS-X), 1% l-Glutamine. PDX305 cells were cultured in IMDM supplemented with 20% FBS, 1% ITS-X, 1% l-Glutamine, and 1% Antibiotic-Antimycotic. All cells were maintained at 37 °C in 5% CO_2_. For encapsulation in hydrogels or seeding on tissue culture plastic, cell density was chosen according to best viability conditions for 3D and manufacturer’s recommendations for 2D. In general, EwS cells were seeded at 10 × 10^6^ cells/mL for encapsulation conditions.

### 2.2. Peptide synthesis and purification

Fmoc-protected amino acids were purchased from ChemPep Inc. (Wellington, FL). Azide (N_3_)-modified peptides (N_3_-GRGDS-NH_2_, N_3_-GDGEA-NH_2_, N_3_-GGFOGER-NH_2_, N_3_-GAEIDGIEL-NH_2_) and the diazide peptide crosslinker [N_3_-RGPQGIWGQLAETGGRK(N_3_)-NH_2_] were synthesized on a Gyros Peptide synthesizer. The N-terminal Fmoc and Lys (Dde) protecting groups were removed on resin prior to dual azide modification. The resin-bound peptide was transferred to a synthesis vessel and rinsed thrice for 10 min in a 15 mL solution containing 2% hydrazine solution in dimethylformamide (DMF). The azides on lysines or N-terminal were conjugated by reacting 4-azidobutanoic acid (2 mmol, 259 mg), O-(7-Azabenzotriazol-1-YL)-N,N,N',N'- tetramethyluronium hexafluorophosphate (HATU, 750 mg, 1.97 mmol) and DIEA (1.38 mL, 4 mmol) in 5 mL DMF for 90 min. Peptide cleavage occurred in 15 mL trifluoroacetic acid (TFA) containing 0.375 mL triisopropylsilane (TIS) and 0.375 mL water. Following cleavage, the crude peptide samples were crashed in ice-cold diethyl ether, pelleted by centrifugation, and dried under nitrogen. The dried product was dissolved in 5% acetonitrile and purified using high-performance liquid chromatography (HPLC). The fractions with the highest peaks were collected and the mass was verified through mass spectrometry. The fractions containing the desired peaks were then lyophilized and stored in −80 °C until further use. All peptides once purified and lyophilized were resuspended either in sterile phosphate buffered saline (PBS) or 20% DMSO in PBS for less soluble peptides.

### 2.3. Hydrogel fabrication

Peptides and their scrambled versions were lyophilized, resuspended in PBS and then stored in the −80 °C until usage. Azide-modified peptides were conjugated to poly(ethylene glycol) tetra-bicyclononyne (PEG-tetraBCN, MW ~ 20 kDa) by SPAAC chemistry. Concentrations of the peptides reflected their physiological distribution. To make ‘RC’ hydrogels, 0.64 mM N_3_-GRGDS-NH_2_, 0.12 mM N_3_-GDGEA-NH_2_, and 0.12 mM N_3_-GGFOGER-NH_2_ were mixed with 3 mM PEG-tetraBCN. The solution was allowed to react in the incubator at 37 °C until ready to be crosslinked. The crosslinker N_3_-RGPQGIWGQLAETGGRK(N_3_)-NH_2_ (6 mM) was mixed with the PEG-peptide solution at a 1:2 molar ratio of PEG:Crosslinker. The entire solution was incubated at 37 °C and 5% CO_2_ for 30 min for gelation to occur. The molecular weights and working concentrations for all peptides are listed in Table 1.

### 2.4. Cell encapsulation in hydrogels

Cells were processed for encapsulation while the PEG-peptide solution reacted in the incubator. Upon counting, a desired cell concentration was mixed with the PEG-peptide solution following which the diazide crosslinker was added to the final mix. The whole solution containing PEG, peptides, cells, and the crosslinker was pipetted directly onto the bottom of 96-well plates or on RainX-treated glass slides with 500 μm spacers. CHLA10 or PDX305 cells were encapsulated as single-cell suspensions in RC/RCT hydrogels at a density of 10 × 10^6^ cells/mL unless otherwise stated and cultured for 5 days. The 5-day timeline is typical to observe TNC secretion by EwS cells [38-40]. Single-cell encapsulations allowed for independent cluster formation of the cells. The size of the hydrogels depended on the application. Cells were encapsulated in 5 μL hydrogels for viability assays and immunocytochemistry. The hydrogel droplet size increased to 20 μL for flow cytometry and 25 μL for qRT-PCR.

### 2.5. Sortase expression and purification

The plasmid to express Sortase A eSrtA (2A-9) (2A9) in pET29b was a gift from David Liu (Addgene Plasmid #75145). Transformed BL21 *E. coli* were grown in 500 mL Luria Broth and induced using isopropyl β-d-1-thiogalactopyranoside (0.5 mM) at an OD600 of 0.6, following which expression was conducted at 18 °C overnight. Cells were lysed by sonication on ice with a 1 min ON, 2 min OFF cycle repeated 6 times. The sonicated solution was centrifuged at 10,000 g for 1 h at 4 °C. The supernatant was collected post centrifugation as this contained the crude 2A9. Sortase 2A9 was then purified by immobilized metal affinity chromatography from the clarified lysate using an ÄKTA Pure 25 L FPLC (Cytiva; Marlborough, MA), spin concentrated, and stored at −80 °C in PBS containing 20% glycerol.

### 2.6. Mass spectrometry

Successful purification of the peptides was confirmed via Electrospray Ionization Liquid Chromatography Mass Spectrometry (ESI-MS) (Fig S1 a-c, Fig S2c, Fig S6a). Mass spectrometry was performed by direct injection of 40 μL purified solution released from the HPLC in glass ampoules. Expected masses were the dominant observed peaks for each sample with an additional −26 peak corresponding to azide reduction to an amine during sample ionization.

### 2.7. CellTiter-Glo

The CellTiter-Glo^®^ Luminescent 3D Cell Viability Assay (Promega, Madison, WI, USA) was performed according to the manufacturer’s instructions. CellTiter-Glo 3D reagent was pipetted in the wells containing hydrogels at a 1:1 ratio with media. Samples were incubated for 30 min at 37 °C. Data was collected by measuring luminescence using a Tecan Fluorescence Plate Reader and analyzed as described in the statistics section.

### 2.8. Live/Dead staining

Cell viability was measured by employing a Live/Dead Viability Kit (L3224, Thermo Fisher, Waltham, MA). Calcein AM marks live cells by detecting cellular esterase activity and emitting a green, fluorescent signal at 517 nm. Dead cells are marked by ethidium homodimer-1 that internalizes in cells with a compromised plasma membrane emitting fluorescence at 617 nm. Cells encapsulated in hydrogels were stained with 2 μM Calcein AM and 4 μM ethidium homodimer-1 in PBS, and incubated at room temperature in the dark for 30 min. Post-incubation, the samples were rinsed 3 times with PBS for 5 min and imaged on a Leica THUNDER Imager 3D Tissue (Leica Microsystems, Wetzlar, Germany) microscope.

### 2.9. AlamarBlue^™^ high sensitivity (HS) cell viability reagent

Cell metabolic activity was measured by AlamarBlue. The AlamarBlue reagent (10%) was directly added to samples in well plates and incubated for 2 h at 37 °C. The supernatant was transferred in an opaque black-bottom plate and fluorescence measured on a Spectral Plate Reader (excitation=535 nm, emission=575 nm). Media was replaced after AlamarBlue measurements. Metabolically active cells convert resazurin to a red fluorescent product resosurfin. The fluorescence levels indicate metabolic activity which is also directly proportional to the number of living cells.

### 2.10. Rheometry

Plateau modulus post gel swelling was measured at 37 °C using 8 mm parallel plate geometry (Gap: 0.5 mm; Strain: 1%; Frequency: 1 Hz) on a Physica MCR301 rheometer (Anton Paar; Graz, AT) for 30 μL hydrogels containing 3 mM PEG-tetraBCN and 6 mM di-azide peptide crosslinker. Briefly, the gels were placed directly onto the bottom plate with the probe slowly lowered to contact the gel. PBS was deposited to the periphery of the gel to avoid gel dehydration. Frequency sweeps were performed to measure storage and loss moduli with frequencies incrementally increasing from 1 Hz to 10 Hz. Data are represented with G’ values at the same frequency.

### 2.11. Sortase treatment for hydrogel degradation and cell retrieval

For downstream analyses, hydrogels were treated with a solution of 50 μM 2A9, 18 mM GGG, and 10 mM CaCl_2_ for 15–30 min with constant agitation at 37 °C. To ensure maximal retrieval of viable cells, two degraded gels per condition were pooled together. The entirety of the degraded hydrogels and cells solution was collected in 1.5 mL Eppendorf sterile tubes and centrifuged at 600 g for 3 min. The supernatant was aspirated and the cell pellet was washed 3X with fresh media to remove any remaining 2A9. The cells were then either lysed to collect RNA for qRT-PCR or processed further for flow cytometry. For conservative usage of polymers and peptides, we identified that two 25 μL hydrogels when pooled provided sufficient RNA to perform several rounds of qRT-PCR and two 20 μL hydrogels pooled was sufficient to conduct flow cytometry. In these instances, the reported n represents multiple biological replicates of pooled gels.

### 2.12. Flow cytometry

EwS cells were retrieved from gels following 2A9 treatment. The cell pellet was dissociated briefly with trypsin following three washes with media to eliminate any remaining sortase. The cells were further resuspended and washed with Fluorescence-Activated Cell Sorting (FACS) buffer containing 2% FBS in PBS. Post washing, the cells were stained with fluorophore-conjugated antibodies for 30 min incubated on ice and protected from light. The antibodies used were FITC Mouse Anti-Human CD99 (#555688), PE Mouse Anti-Human CD44 (#550989), and PE Mouse IgG1 K Isotype control (#555749) all purchased from B.D. Pharmingen.

### 2.13. qRT-PCR

For RNA isolation from cells in 2D, lysis buffer (Qiagen) was directly added to the wells. For isolating RNA from 3D retrieved cells, lysis buffer was added to cell pellet after sortase degradation. Equal proportion of 70% ethanol was added to the samples and up to 700 μL of the solution was transferred to collection tubes. RNA isolation and purification was performed using manufacturer’s instructions. For uniformity in input, total RNA was normalized to the minimum concentration amongst the samples with 260/280 value of approximately 2.0. Complementary DNA was synthesized using iScript cDNA Synthesis Kit (Bio-Rad) according to manufacturer’s instructions. qPCR was performed using iTaq Universal SYBR-Green Supermix (Bio-Rad) on a Roche Light-Cycler 480 instrument (Roche Applied Science) in the presence of primer sequences (IDT) using the cycling parameters as follows: 95 °C for 3 min (denaturation), 95 °C for 3 s, 60 °C for 30 s for 45 cycles. The genes used for PCR were *CD44, MKI67, MMP9, MMP2, LGMN, ITGA9, ITGB1, CALD1, TNC*, and *COL1A1;* the housekeeping genes were *18S* and *EEF1A1*. The primer sequences are as follows: *CD44* forward: GATGGAGAAAGCTCTGAGCATC, *CD44* reverse: TTGCTGCACAGATGGAGTTG; *MKI67* forward: AAAAGAATTGAACCTGCGGAAG, *MKI67* reverse: AGTCTTATTTTGGCGTCTGGAG; *MMP9* forward: CATTCAGGGAGACGCCCATT, *MMP9* reverse: AACCGAGTTGGAACCACGAC; *MMP2* forward: ATGGGGAATCGGTTGAAGG, *MMP2* reverse: AATTGCATTTCCTGACAGAAGG, *LGMN* forward: CCTGAAGATGGAGGCAAGCACT, *LGMN* reverse: GTTCGTCAGGAATCCCATTGCG, *ITGA9* forward: TCAGGTCACAGAGAAGCTGCAG, *ITGA9* reverse: CACATGCTCACTGAGGCTGTAG, *ITGB1* forward: GGATTCTCCAGAAGGTGGTTTCG, *ITGB1* reverse: TGCCACCAAGTTTCCCATCTCC, *CALD1* forward: TCTGAGCCTTCTGGTTGGTC, *CALD1* reverse: CCTCGGGAAGAAGTTTCAGA, *TNC* forward: GCAGCTCCACACTCCAGGTA, *TNC* reverse: TTCAGCAGAATTGGGGATTT; *COL1A1* forward: CTGGACCTAAAGGTGCTGCT, *COL1A1* reverse: GCTCCAGCCTCTCCATCTTT; *18S* forward: GCAATTATTCCCCATGAACGA, *18S* reverse: GGCCTCACTAAACCATCCAAT; *EEF1A1* forward: CGTTACAACGGAAGTAAAATC, *EEF1A1* reverse: CAGGATAATCACCTGAGC. Samples were run in triplicates and average Ct values were normalized to the geometric mean of both housekeeping genes. Relative mRNA expression was calculated using the ΔCt method.

### 2.14. Immunofluorescence staining

All samples were fixed with 4% paraformaldehyde for 45 min followed by rinses with tris-buffered saline (TBS) at room temperature (RT) on a shaker. Permeabilization occurred with 0.5% Triton-X in TBS for 45 min followed by TBS rinses. Samples were blocked in 1% BSA overnight at 4 °C. Primary antibodies were diluted in 0.5% BSA and incubated overnight at 4 °C. The primary antibodies used were TNC (1:1000, #MA126779, Invitrogen), pSRC(Y419) (1:100, #44–660G, Invitrogen), Rabbit IgG (1:100, #ab172730, Abcam), Mouse IgG (1:100, #ab18443, Abcam). An additional antibody for integrin α9β1(Y9A2) (5 μg/mL, #MCA1585T, BioRad) was used for integrin blocking experiments. Alexa-fluor conjugated secondary antibodies were either incubated overnight or for at least 3 h in 0.5% BSA at 4 °C in the dark. The secondary antibodies were Goat Anti-Mouse Alexa Fluor 555 (1:200) and Goat Anti-Rabbit Alexa Fluor 568 (1:200). Directly conjugated Ki67-Alexa Fluor 647 (1:200, #4414, Cell Signaling) was applied during the secondary incubation. Nuclei were stained using 1 μg/mL DAPI which was included during secondary antibody incubations. Upon adding secondary antibodies, samples were rinsed thrice with TBS and imaged on a Leica THUNDER Imager 3D Tissue (Leica Microsystems, Wetzlar, Germany) microscope.

### 2.15. Imaging and image analysis

Imaging was performed with a Leica THUNDER microscope at 20X or 40X magnification. Images were captured with the same exposure and gain settings within each data set. At least three hydrogel replicates (5 images per hydrogel) were used per condition unless otherwise stated. Z-stacks (350 μm) were captured across various areas for each hydrogel. Images were processed for background subtraction using the Instant Computational Clearing (ICC) method provided with the Leica LASX software. Images were imported into FIJI for quantification of TNC expression via measuring the mean fluorescence intensity (MFI) of each field of view after splitting channels. To quantify positive signal for cellular markers such as Live/Dead, Ki67, pSRC, channels were split into 8-bit grayscale images. The split images were thresholded and cells were counted using FIJI’s ‘automated cell counting of single-color image’ feature. For multicellular clusters, images were processed using ‘watershed’ on FIJI before counting the cells. If clusters were challenging to separate even after watershedding, the cluster numbers were counted using the same methods in addition to defining circularity of clusters. All cellular markers being assessed were normalized to total DAPI^+^ cells for comparison between groups. Quantification of the Live/Dead staining was performed similarly, except the total number of dead and live cells was considered as the denominator of ‘total cell count’ to calculate ratios of live cells/total cells and dead cells/total cells. All data were exported to GraphPad Prism for further statistical analyses.

### 2.16. Statistical analysis

Statistical analysis was performed using GraphPad Prism v9/10. For samples with equal variance, the significance between groups was analyzed using a two-tailed student’s *t*-test. For samples with unequal variance, a two-tailed Welch’s *t*-test was performed. For multiple comparisons and grouped comparisons, a one-way ANOVA or two-way ANOVA with post hoc Tukey's test or post-hoc student’s *t*-tests was used. The results were deemed significant at 0.01 < *P ≤ 0.05 and 0.001 < **P ≤ 0.01, highly significant at ***P ≤ 0.001, and extremely significant at ****P ≤ 0.0001. In general, the sample sizes involve n = 3–6.

## 3. Results

### 3.1. Cancer cells require adhesive peptides for survival and growth in ECM-mimicking hydrogels

**Fig. 1.**
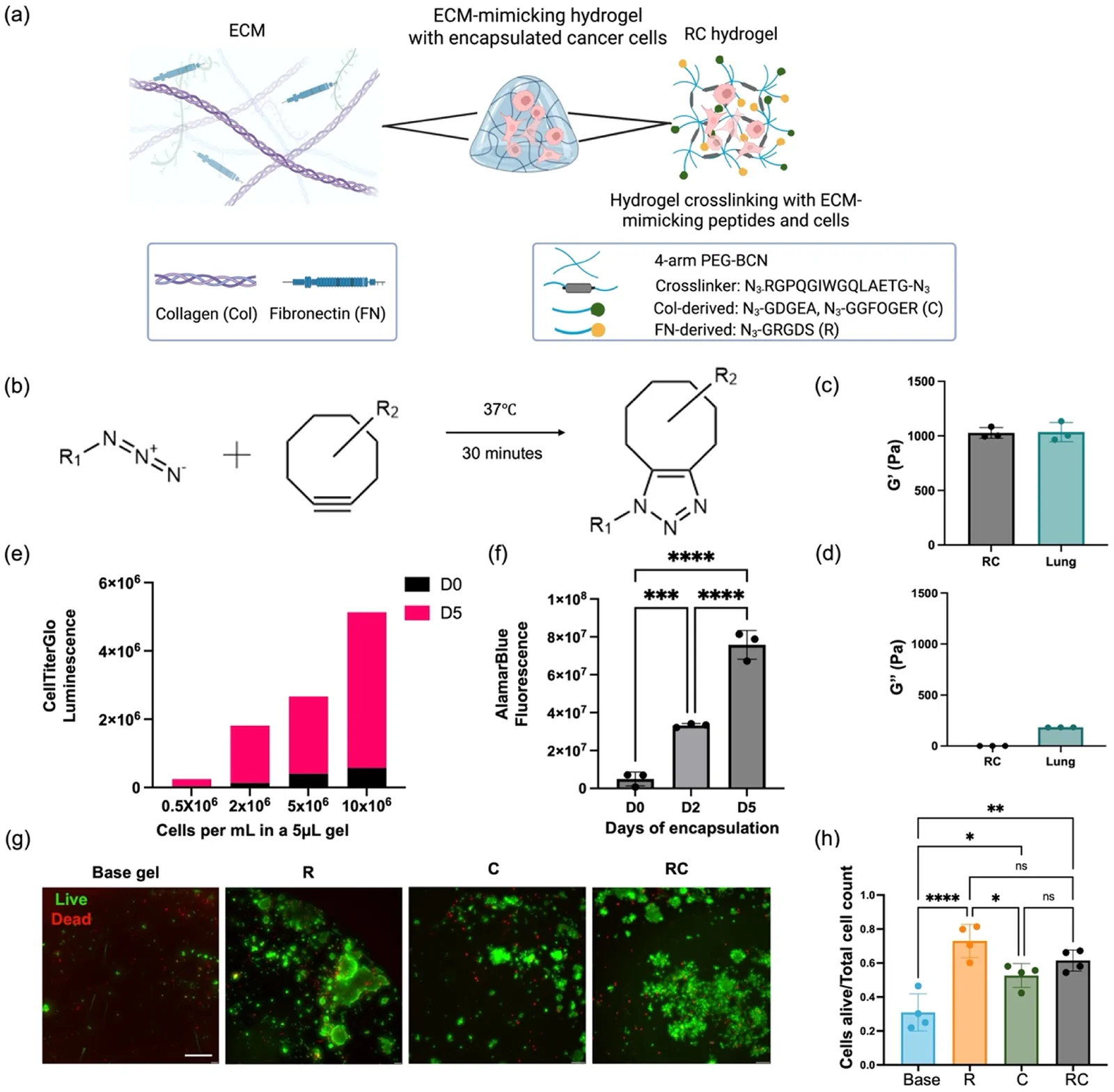
RC hydrogels promote cancer cell viability and growth. (a) Schematic for the ECM-mimicking RC hydrogel with a 4-arm PEG-tetraBCN scaffold as the base, azide-modified ECM peptides as pendants, and an MMP/SrtA-degradable diazide peptide crosslinker. (b) SPAAC reaction between azide and BCN groups drives hydrogel polymerization. (c) The stiffness of RC hydrogels (as a measure of storage modulus G’) is in the same range as that of a lung around 1000 Pa. (d) Loss modulus of RC is very low, as expected for a predominantly elastic material. (e) CellTiter-Glo measurements to quantify viability of EwS cells encapsulated at increasing density within RC gels. EwS cell growth is affected by encapsulation density and is fastest with 10 × 10^6^ cells/mL per 5 μL gel. (f) AlamarBlue measurements of 10 × 10^6^ cells/mL in RC gels to measure metabolic activity depict an increase in fluorescence from day 0 to 5 post-encapsulation. (g) Live/Dead staining of EwS cells in various combinations of the hydrogels – Base gel lacking ECM peptide, R, C, RC. Scale bar = 100 μm. Cells did not survive encapsulation without addition of bioactive adhesion-mediating peptides. (h) Highest viability was observed in R and RC hydrogels making ‘RC’ hydrogels the optimal design to mimic the ECM. One-way ANOVA, Tukey’s post-hoc, n = 4, *p < 0.05, **p < 0.01, ***p < 0.001, ****p < 0.0001.

Hydrogels were synthesized via strain-promoted azide-alkyne cycloaddition (SPAAC) click chemistry between a 4-arm PEG-tetraBCN (3 mM) backbone (Fig. 1a,b) and a diazide peptide crosslinker N_3_-RGPQGIWGQLAETGGRK(N_3_)-NH_2_ (6 mM), with gelation completed in 30 min at 37 °C [41,42]. The peptide crosslinker was designed to feature both a matrix metalloproteinase (MMP)-sensitive motif (PQGIWGQ) to promote in situ matrix remodeling of encapsulated tumor cells, and a SrtA-cleavable sequence (LAETG) for user-controlled degradation and recovery of encapsulated cells for downstream analyses.

To create biomaterials that mimic the biochemistry of the solid TME, we modified the base hydrogel formulation with integrin-binding monoazide peptides from the most abundant ECM proteins found in these tissues *in vivo* [43], including FN and Col [33-36]. RGDS was included as an FN mimic, as was Col-derived DGEA and GFOGER (Fig. 1a); two Col-derived peptides were included to engage multiple integrins on metastatic cancer cells. Mass spectrometry of the purified peptides is shown in Fig. S1a-c. Peptide ratios were selected to match those comprising the soft tissue lung TME [44], with 0.64 mM RGDS, 0.12 mM DGEA, and 0.12 mM GFOGER peptides. Gels containing RGDS are denoted as “R”; those with Col peptides are referred to as “C”; those with both are indicated as “RC” gels (Fig. 1a).

Hydrogel mechanics were measured through rheological assessments. Quantifying storage and loss moduli (G’ and G") of the base, R, and C hydrogels suggests no significant difference in stiffnesses to RC hydrogels, indicating that any observed biological variations are a result of differing biochemical cues and not biophysical cues (Fig S1d,e). To keep stiffness and crosslinker density consistent between all hydrogels, the total peptide concentration was kept constant; gels lacking a bioactive peptide were formulated with a matched concentration of their scrambled counterpart. Hence, the storage modulus (approximately 1 kPa) for the RC hydrogel was similar to the hydrogels without ECM-peptides. Beyond this, the G’ values for RC hydrogels are statistically indistinguishable from that experimentally determined for healthy murine lung (Fig. 1c,d) with the G” value being nearly 0 for RC hydrogels. The stiffness of a murine lung is also not statistically different from a human lung [45]. The rheological measurements support the design of an ECM- or soft tissue-mimicking 3D hydrogel.

To verify functionality of the hydrogels as mimics for a soft tissue metastatic TME, we investigated the biological and functional alterations in encapsulated EwS cancer cells. We focused our studies on CHLA10, an established EwS cell line that secretes high levels of TNC [46]. Key experiments were repeated with PDX305 cells, a second TNC-producing EwS cell line that was derived from an early passage patient-derived xenograft [40,47]. Both cell lines were originally sourced from resected soft tissue metastases from patients with recurrent/refractory EwS. The CHLA10 model was derived from an adolescent female while PDX305 tumor was from an adolescent male. CHLA10 cells were encapsulated in the RC gel at four different seeding densities (0.5 × 10^6^, 2 × 10^6^, 5 × 10^6^, 10 × 10^6^ cells per mL for a 5 μL gel). Viability was measured via CellTiter-Glo Luminescent Cell Viability Assay at the day of encapsulation (day 0) and at end point (day 5). The results demonstrate that EwS cells were able to grow at all seeding densities in a cell dose-dependent manner (Fig. 1e). The CellTiter-Glo reagent effectively diffused through the hydrogels and luminescence was detected with as few as 2500 cells (0.5 × 10^6^ cells/mL). Assessing metabolic activity from days 0 to 5 through AlamarBlue assay demonstrates a significant increase in cell expansion over time (Fig. 1f).

Next, we sought to understand the role of each isolated peptide in EwS cell viability and growth promotion. CHLA10 cells were grown in each of the gel formulations for 5 days prior to Live/Dead viability analysis (Fig. 1g). Cell survival in hydrogels lacking adhesive peptides or with a scrambled RGDS peptide (N_3_-GRDGS-NH_2_) was poor, suggesting that EwS cells require adhesion, through RGDS for example, in 3D microenvironments to survive (Fig S1f,g). While C gels also promoted survival, more cells died compared to the R condition (Fig S1h). The greatest viability was observed in R and RC gels (Fig. 1g,h), with no significant differences observed between these conditions. Since RC gels more accurately simulate the ECM composition of a lung TME due to inclusion of FN- and Col-derived peptides, RC hydrogels were chosen as the baseline lung ECM-mimicking hydrogel design for subsequent studies.

### 3.2. Sortase-mediated degradation enables downstream analyses and is not detrimental to cell health

**Fig. 2.**
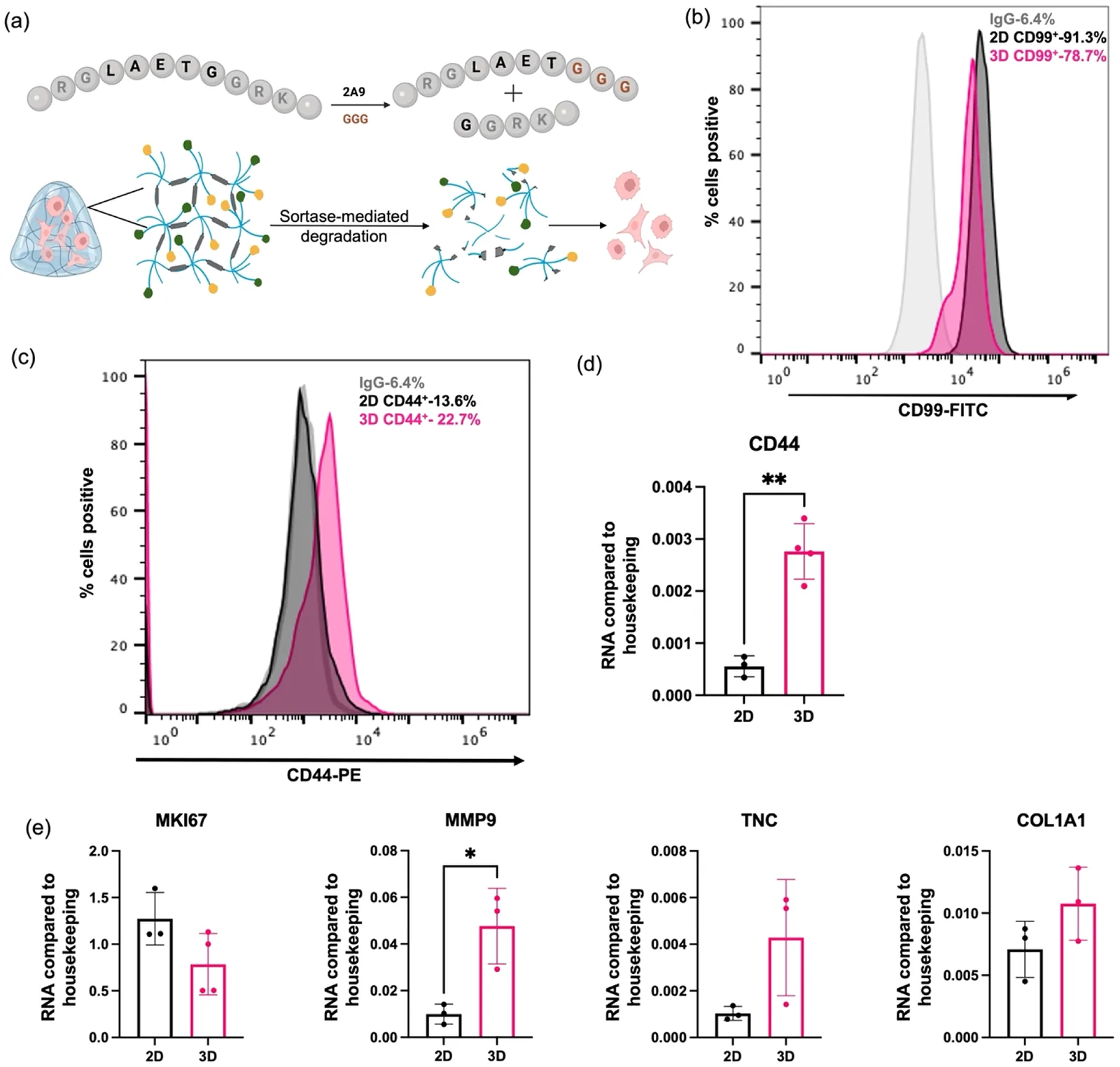
ECM-mimicking hydrogels are designed for optimal cell recovery using sortase-mediated degradation. (a) Schematic for the crosslinker with the sortase substrate motif ‘LAETG’ wherein 2A9 breaks down the crosslinked hydrogel network releasing the encapsulated cells. (b) CD99 expression of EwS cells cultured on 2D tissue culture plastic and in 3D RC hydrogels measured by flow cytometry post-sortase degradation. The x-axis represents the intensity of FITC-conjugated CD99 expression and the y-axis represents the percentage of positive cells. A shift to the right indicates an increase in the % positive cells. EwS cells in 3D and 2D were both CD99^+^. Quantifying CD44 expression through (c) flow cytometry as well as (d) qRT-PCR indicates an increase in CD44 expression in 3D compared to 2D (e) Gene expression for proliferation marker MKI67 is modestly decreased in 3D, while matrix remodeling gene (MMP9) is significantly increased and ECM-related genes (TNC, COL1A1) are higher in 3D than 2D. Student’s *t*-tests, n = 3 or 4, *p < 0.05, **p < 0.01.

A potential challenge with using synthetic hydrogels for 3D studies is the retrieval of encapsulated cells for downstream analyses. Low yields and quality of RNA, disruption to cell membranes, and ultimately cell death present difficulties in cell recovery from 3D platforms [48]. To mitigate this obstacle, the hydrogels were designed for recovering encapsulated cells in a “biologically invisible” manner for downstream functional and mechanistic analyses via sortase transpeptidation [22]. Here, purified sortase 2A9 (Fig S2a,b) catalyzes a transpeptidation reaction between triglycine and its LAETG peptide substrate (peptide’s mass shown in Fig S2c), yielding rapid gel breakdown and cell release (Fig. 2a). We performed qualitative and quantitative assays including viability assays, gene expression, and flow cytometry of retrieved cells to ensure retention of cell surface receptors and high yields and integrity of RNA (Fig S2d). The gating strategy for live cells is shown in Fig S3. Furthermore, we performed hydrogel stability measurements to ensure no leaching or gel breakdown occurred without external stimulus (Fig S4). Images of hydrogels before and after crosslinking, as well as pre- and post-sortase degradation, are in Fig S4a-e. Hydrogels were cast with a fluorescently labeled azide and fluorescence of crosslinked hydrogels, sortase-degraded gels, and soluble fluorescent azides were measured over a week. Minimal fluorescence was detected in the crosslinked hydrogels with a significant increase in fluorescence of degraded hydrogels (Fig S4f). The absence of supernatant fluorescence from hydrogels sampled over 7 days indicates that this is a stable crosslinked network with minimal degradation or leaching. Mesh size of these networks, calculated using storage moduli (calculations in SI), was around 16–16.6 nm which is large enough for diffusion of small proteins and growth factors.

Once recovered from the RC hydrogels using 2A9, CHLA10 cells were fluorescently stained for CD99, a positive cell surface marker of EwS, and expression measured by flow cytometry. The results show that CD99 remains intact on recovered CHLA10 cells following 2A9 treatment and retrieval [Mean ± Standard deviation (M±S.D.) = 78.7%±1.4], albeit at a somewhat lower level than when the same cells are cultured in 2D (91.3%±0.1) (Fig. 2b). Whether the differences between detection of CD99 in 2D vs. 3D conditions represent technical or biologic effects is not known. Nevertheless, these results confirm that flow cytometry-based analysis of retrieved tumor cells is feasible. CD99 expression was also measured with PDX305 cells and a similar trend as CHLA10 cells was observed (Fig S5a). To our knowledge, this is the first demonstration that sortase-based degradation is compatible with antibody-based detection of surface receptors.

Next, we used flow cytometry to measure CD44 expression on retrieved cells. CD44 is a less ubiquitously expressed surface protein that plays a key role in mediating tumor: ECM adhesion and promoting EwS cell migration and invasion [49]. Significantly, the percentage of CHLA10 EwS cells expressing surface CD44 increased in 3D RC hydrogels (22.7% ± 2.1) compared to 2D tissue culture plastic (13.6% ± 0.3) (Fig. 2c). CD44 was nearly universally expressed by PDX305 cells in both 2D and 3D cultures (Fig S5b) [32].

Having established that tumor cells retrieved from RC hydrogels can be assessed by flow cytometry, we next evaluated recovery of RNA. Cells were retrieved from RC gels using 2A9 treatment and RNA was isolated immediately. High quality RNA was reproducibly obtained as evidenced by 260/280 values of ~2.0, yields of greater than 110 ng/μL, as well as RNA integrity numbers (RIN) between 9.5–10 for all samples (Fig S2d). We then performed qRT-PCR for ECM-related genes and other markers of interest. Consistent with the increase in CD44^+^ cells by flow cytometry, expression of *CD44* transcript was increased in CHLA10 cells recovered from 3D RC hydrogels compared to 2D tissue culture plastic (Fig. 2d).

We next analyzed the expression of standard markers of proliferation and of matrix associated genes to assess the impact of the 3D gels on cell states (Fig. 2e). The proliferation marker *MKI67* was relatively downregulated in 3D, whereas the matrix remodeling enzyme-encoding gene *MMP9* was significantly increased. While ECM protein-encoding genes *TNC* and *COL1A1* were not significantly different between 2D and 3D, the trends indicate an increase in ECM-related gene expression when EwS cells are cultured in 3D hydrogels.

Together these data confirm that cancer cells can be successfully encapsulated within and subsequently retrieved from, synthetic 3D RC hydrogels. Feasible downstream biologic assays include measurements of cell viability, cell surface marker expression by flow cytometry, and high yield and quality RNA isolation for gene expression studies. More importantly, differences in cell biology are evident between 2D tissue culture plastic and 3D ECM-mimicking hydrogels that are likely to be relevant to physiologic interactions between a tumor and its lung metastasis niche TME.

### 3.3. RC hydrogels can be tuned to include a TNC-derived peptide, mimicking a key feature of metastatic niches

**Fig. 3.**
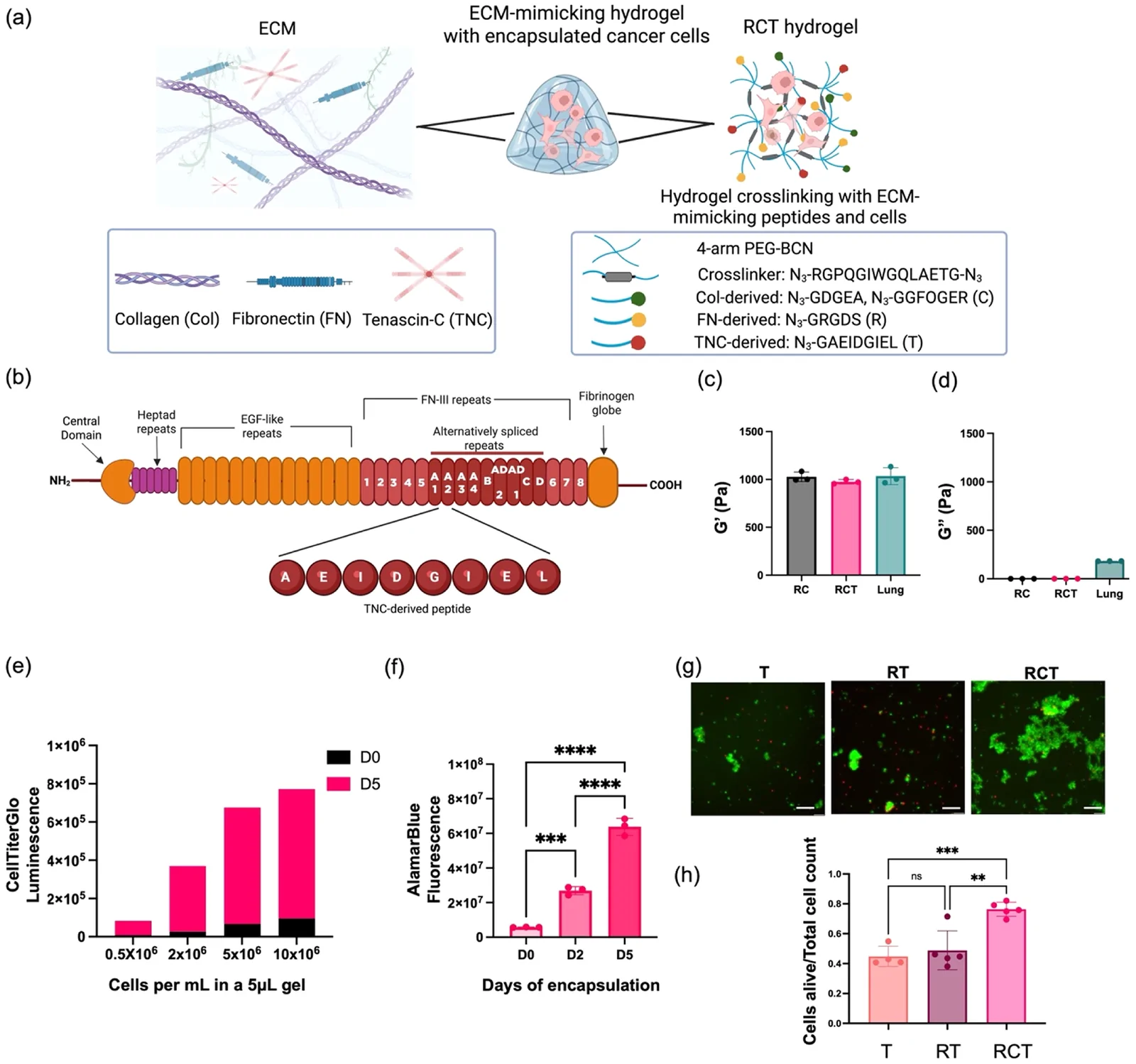
Addition of the TNC-derived peptide does not affect gel stiffness or cancer cell viability. (a) Schematic for RCT hydrogel incorporating the aforementioned peptides in the RC hydrogel along with the TNC-derived peptide AEIDGIEL. (b) Representation of the TNC protein demonstrating the isolation of the TNC-derived peptide from the FN-III repeats. Both RC and RCT hydrogels exert similar mechanical properties and share stiffness levels with the lung as demonstrated by (c) similar storage moduli (G’) and (d) nearly 0 loss moduli. RC and Lung’s G’ and G” values are previously shown in Fig. 1. (e) CellTiter-Glo measurements of increasing cell numbers encapsulated in RCT hydrogels. (f) AlamarBlue measurements of 10 × 10^6^ cells/mL in RCT hydrogels from day 0 to 5 post-encapsulation depict an increase with time. (g) Live/Dead staining for EwS cells in T, RT, RCT hydrogels. Scale = 100 μm (h) Quantification of the live/dead demonstrates highest viability in RCT hydrogels vs. T or RT hydrogels. One-way ANOVA, Tukey’s post-hoc, n = 3 or 4, **p < 0.01, ***p < 0.001, ****p < 0.0001.

The TNC glycoprotein has been identified as an abundant component of the ECM of many solid tumor metastases in the lung [43]. Previous work from our group has highlighted the importance of TNC to the pathobiology of EwS and shown it to be spatially heterogeneous within the TME [31]. Therefore, we tuned the RC hydrogel to simulate a more complex TME by including an azide-modified TNC-derived peptide AEIDGIEL (Fig. 3a) (Fig S6a). This peptide is derived from FN-III repeat domains of TNC that bind to integrin α9β1 (Fig. 3b) [50] and is implicated in cancer cell growth, metastasis, and invasion [51]. Gels containing only the TNC functional peptide are denoted “T”; those additionally containing RGDS are referred to as “RT”; RC gels further supplemented to contain the TNC peptide are denoted as “RCT”.

Rheological measurements revealed that both RC and RCT hydrogels have similar viscoelasticities (Fig. 3c,d) (Fig S6b,c). To ensure no changes in bulk mechanical properties occurred after incorporation of T, a swelling test was performed for RC and RCT (Fig S6d,e). There was no statistically significant difference between the swelling ratio of RC (5.6 ± 0.8) and RCT (4.1 ± 0.3) (Fig S6d). Similarly, no differences were observed in the hydrogel water content between RC (87%±7.3) and RCT (84%±13.2) (Fig S6e). These data indicate that network mechanics are unaltered by TNC peptide addition. Considering the importance of physical properties like stiffness in exacerbating metastatic potential of sarcoma cells [52], these results indicate that any gel-dependent changes in cell function are a direct consequence of the biochemical signaling from the TNC peptide (Fig. 3c).

To assess whether addition of T to the hydrogels influenced cancer cell viability, we encapsulated CHLA10 cells at four seeding densities and performed CellTiter-Glo measurements as stated above. Similar to trends observed with RC hydrogels (Fig. 1e), the RCT hydrogel supported growth of CHLA10 cells in a cell dose-dependent manner (Fig. 3e). AlamarBlue metabolic measurements increase over time indicating successful growth of metabolically active cells in RCT hydrogels (Fig. 3f). Viability, assessed by Live/Dead staining was higher in RCT than T or RT hydrogels (Fig. 3g,h, Fig S6f). These data showcase that inclusion of the TNC peptide does not alter the biophysical properties of the hydrogel. In addition, they reveal that EwS cells can be successfully propagated within this 3D biomimetic matrix that is engineered to incorporate a key ECM component of metastatic niches.

### 3.4. Addition of TNC peptide to RC hydrogels alters EwS cell behavior

**Fig. 4.**
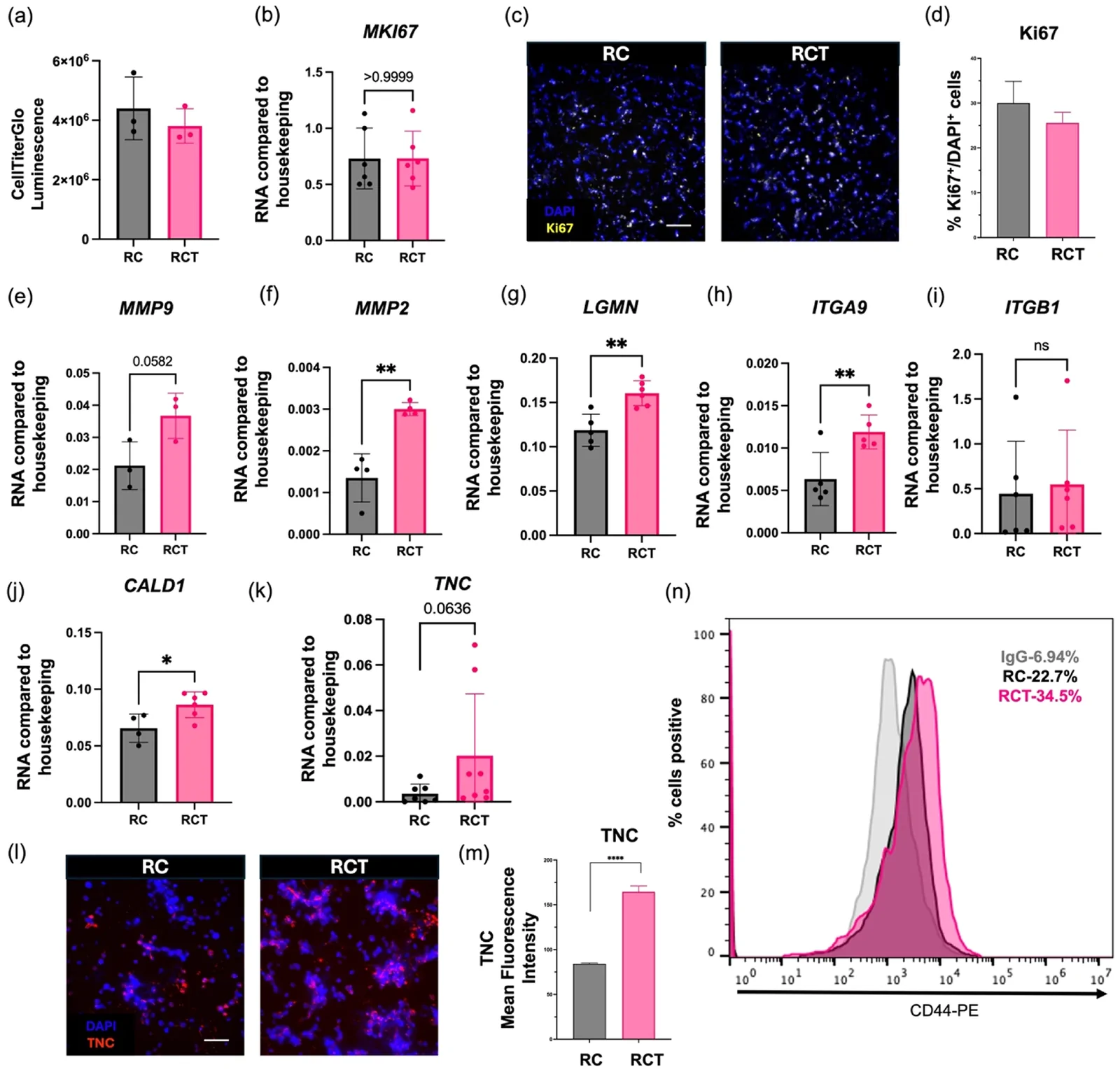
Addition of the TNC peptide alters biologic features of EwS cells. (a) CellTiter-Glo measurements indicate no difference in viability between RC and RCT hydrogels. No significant differences in proliferation observed through (b) gene expression of *MKI67* and through (c) fluorescent staining for Ki67. Cells are stained with DAPI (nuclei; blue) and Ki67 (yellow). (d) Quantification of Ki67 by %Ki67^+^/DAPI^+^ cells is unchanged between RC and RCT. Matrix remodeling genes (e) *MMP9* and (f) *MMP2*, (g) *LGMN*, and cell state transition-associated genes (h) *ITGA9*, (i) *ITGB1*, (j) *CALD1*, and (k) *TNC* in RC versus RCT gels. *MMP2, LGMN, ITGA9*, and *CALD1* are significantly upregulated in RCT gels; *MMP9* and *TNC* trend higher; *ITGB1* is unchanged. (l) Addition of the TNC peptide to the hydrogel augments TNC expression by tumor cells through immunostaining. Images represent Z-projections of a 350 μm Z-stack through the hydrogels. Cells are stained with DAPI (nuclei; blue), TNC (red). The antibody for TNC only recognizes the whole TNC protein and not the TNC peptide. Scale bar=100 μm. (m) Quantification of TNC expression by MFI. TNC deposition is significantly augmented in RCT hydrogels. (n) Flow cytometry shows a modest increase in CD44-expressing tumor cells in RCT compared to RC hydrogels. Student’s *t*-test, n = 3 or 4, *p < 0.05, **p < 0.01, ****p < 0.0001.

Having created tunable hydrogel compositions simulating both TNC-poor and TNC-rich ECM niches, we next sought to assess if addition of the TNC peptide had an impact on tumor cell phenotype. First, we used CellTiter-Glo assays to examine cell viability in RC and RCT gels. Cell growth after 5 days in culture was found to be equivalent in the RC and RCT hydrogels (Fig. 4a). Additionally, there was no difference in metabolic activity between RC and RCT as measured by AlamarBlue assay (Fig S6g). Equivalent proliferation between the two models was further supported by the observation that expression of Ki67 was unchanged at the level of both RNA (*MKI67)* (Fig. 4b) and protein (Fig. 4c, d). Viability (Fig S7a,b), metabolic activity (Fig S7c), and proliferation (Fig S7d-f) of encapsulated PDX305 cells also showed no differences between RC and RCT gels.

EwS cells are highly plastic and undergo cell state changes in response to cues from their tumor microenvironment [40,53]. Therefore, we next assessed whether addition of the TNC peptide induced morphogenic rather than mitogenic effects. Significantly, multiple genes associated with more mesenchymal EwS cell states were increased in RCT vs. RC hydrogels (Fig. 4e-k). Expression of *MMP2, LGMN, ITGA9*, and *CALD1* was significantly upregulated, while notable trends to increased expression were detected in *MMP9* and *TNC*. Consistent with induction of more mesenchymal cell states, CHLA10 cells cultured in RCT hydrogels upregulated expression of TNC protein (measured by MFI) and the frequency of cells expressing cell surface CD44 also increased relative to RC gels (Fig. 4l-n). PDX305 cells likewise upregulated TNC in RCT gels (Fig S7g-i). This is consistent with prior reports from our group which identified the existence of a TNC-mediated, feed-forward signaling loop in EwS whereby TNC in the microenvironment stimulates EwS cells to secrete more TNC [54].

Together these studies demonstrate that synthetic 3D RC hydrogels can be successfully adapted to incorporate a TNC peptide. Importantly, while this additional tuning of the platform has no impact on tumor cell viability or proliferation, it does induce morphogenic changes to EwS cells consistent with cells adopting a more mesenchymal phenotype. These changes at least partially phenocopy the effects of the full-length TNC protein.

### 3.5. RCT hydrogels upregulate SRC signaling in encapsulated EwS cells

**Fig. 5.**
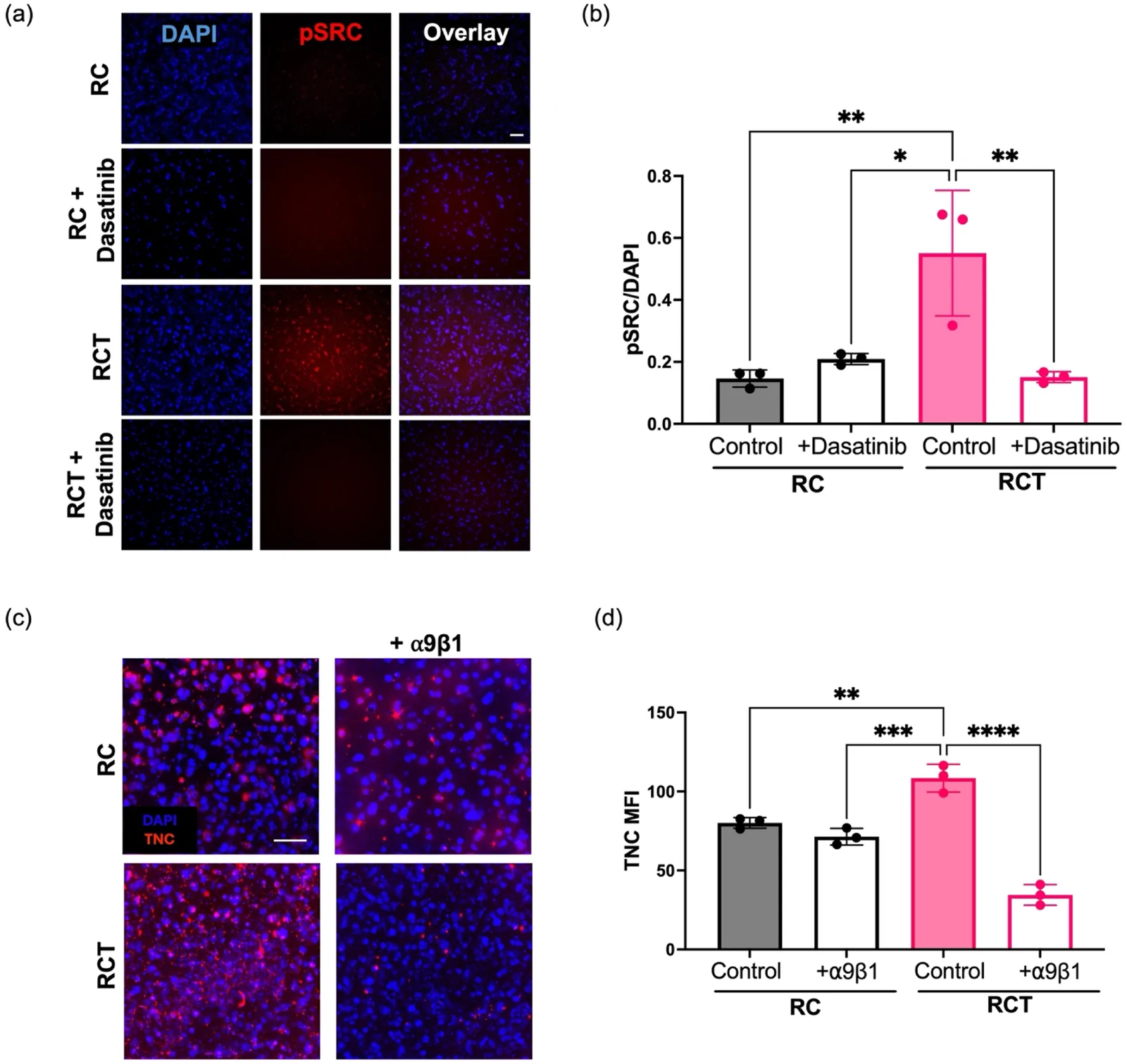
Addition of TNC peptide to RC hydrogel induces SRC activation in EwS cells. (a) Immunostaining for the nucleus (DAPI in blue) and pSRC (red) in RC, RCT hydrogels with and without 10 μM dasatinib treatment. From left to right, the images are represented with their DAPI-blue channel, pSRC-red channel, and an overlay of both channels. Scale bar=100 μm. (b) Quantification of pSRC expression normalized to DAPI^+^ cells demonstrates a significant increase in RCT hydrogels that is completely blocked by the addition of dasatinib. (c) Staining for TNC (red) secreted by CHLA10 cells in RC and RCT with and without blocking α9β1 (5 μg/mL). Images are each Z-projections of 350 μm Z-stacks through the gels. Scale bar = 100 μm. (d) Quantification of TNC expression through MFI demonstrates no effect in RC but a significant downregulation of TNC upon blocking α9β1 in RCT. One-way ANOVA with Tukey’s post-hoc; n = 3, *p < 0.05, **p < 0.01, ***p < 0.001, ****p < 0.0001.

As mentioned above, we previously showed that TME and tumor cell-derived TNC activate a feed-forward signaling loop in EwS that induces cells to deposit more TNC and adopt invasive phenotypes, likely promoting tumor metastasis [31,54]. We therefore investigated if the observed morphogenic effects of the TNC-derived peptide in the RCT hydrogel are mediated by activation of proto-oncogene tyrosine-protein kinase Src (Src). CHLA10 cells were encapsulated in RC and RCT gels and then stained for phosphorylated Src (pSRC) after 5 days. While pSRC^+^ tumor cells were rare in RC gels, pSRC expression increased significantly when cells were encapsulated in RCT gels (Fig. 5a, b). The expression of pSRC was completely lost when cells were exposed to the SRC inhibitor dasatinib, confirming the specificity of the SRC activation signal uniquely in RCT hydrogels (Fig. 5a, b). pSRC activation by RCT was reproduced in PDX305 cells (Fig S7j,k). These data demonstrate that EwS cells show minimal SRC activation in 3D RC hydrogels. By contrast, addition of the TNC peptide robustly activates SRC signaling in EwS cells in the context of RCT hydrogels.

Considering that the TNC peptide binds to integrin α9β1, we used a monoclonal antibody to block α9β1 and evaluated its effect on TNC secretion. Consistent with above, TNC secretion was upregulated in RCT gels and this was blocked by the α9β1 antibody (Fig. 5c, d). These results demonstrate that the TNC peptide induces EwS cells to upregulate TNC expression via direct engagement of the α9β1 integrin receptor and that blocking peptide-receptor binding abrogates this morphogenic response.

Together these studies validate RC and RCT hydrogels as biologically relevant models in which to study interactions between tumor cells and their ECM. They further corroborate published work on the impact of TNC on EwS cell phenotypes. The results presented here reveal the power of utilizing biochemically tunable hydrogels with triggerable degradation as *ex vivo* models of tumor niches.

## 4. Discussion

Biochemical signaling through the ECM is widely recognized as a crucial driver of tumor heterogeneity which contributes to treatment resistance and metastasis [55,56]. A better understanding of cancer cell-ECM interactions is needed to bridge the gap between fundamental biological learnings of ECM ligands and how they influence cancer cell phenotypes and tumor behavior. This work addresses the need for better *ex vivo* models by introducing a highly tunable 3D hydrogel platform designed to mimic the biochemical and biophysical properties of a soft tissue metastatic TME.

Engineered hydrogel systems offer a distinct advantage by allowing the precise, independent tuning of both the biochemical and biophysical properties of the matrix. This level of control can advance our understanding of cell-matrix crosstalk in a way that traditional models cannot [57,58]. For example, while current methods utilize patient-derived spheroids, organoids or “patient avatars,” [59] these platforms often fail to capture the interface between cells and their specific ECM, or they rely on rigid, non-physiological materials (like some alginate or polycaprolactone systems) that do not emulate native soft tissue composition.

Our multi-component hydrogel system (RC/RCT) is designed to overcome these limitations by incorporating enzyme-sensitive linkers and bioactive peptides (representing key soft-tissue ECM proteins) on a PEG scaffold, enabling researchers to build functionality as needed. Unlike bone-mimicking models, our PEG-based hydrogels better emulate soft tissues such as the lung where metastasis predominantly occurs. Rather than whole proteins, we have validated the utility of peptides that are representative of the highly abundant native proteins. Additionally, the SPAAC chemistry utilized here affords a more molecularly uniform polymer network compared to those stemming from chain growth polymerization. This design ensures low batch-to-batch variability, enables anchorage dependence for sarcoma cells, and allows cells to remodel the matrix, traits necessary for sarcoma cell growth and progression of a tumor. Apart from simulating an *in vivo*-like environment, our hydrogels also incorporate a “biologically invisible” sortase-mediated degradation approach seamlessly recovering encapsulated cells without affecting their functional state. Ultimately, the use of advanced *in vitro* models, often referred to as New Approach Methodologies (NAMs), aligns with the push from federal agencies (e.g., FDA and NIH) to reduce reliance on animal models, imparting a major impact on accelerating cancer discovery science and clinical translation.

The RC hydrogels were designed as a reductionist mimic of the native soft tissue microenvironment, leveraging Kundu et al.’s [44] work where they characterized healthy and metastatic lung ECM. We utilized these data to biomimic the peptide compositions and calculate total concentrations for each peptide while maintaining the total grafting capacity of 2 mM for pendant peptides. For example, FN-derived RGDS was 32% of the total concentration that is 0.64 mM, while Col-derived peptides such as GFOGER were 6% which was 0.12 mM to make 2 mM maximum gels. While it was originally known that RGDS was an FN-derived peptide, recent evidence has shown the overlap of the RGD domain with several other ECM proteins including collagens, fibrillin, vitronectin, etc. In essence, a higher presence of the RGDS domain in the hydrogel serves as a moiety that interacts with more than one ECM protein that the cancer cells produce. Additionally, we included two different adhesive Col-derived peptides, DGEA and GFOGER for higher integrin engagement, since both are derived from different regions of the Col protein [60-62]. The hydrogels exhibit similar viscoelastic and bulk rheological properties. While the stiffnesses of the hydrogels are comparable to lung, it is possible to alter stiffness to exclusively look at changes in cancer cell state due to biophysical effects rather than biochemical signals. Ultimately, the hypothesis-driven design of the base hydrogel led to RC hydrogels being a reductionist mimic of the native soft tissue microenvironment focusing on the ECM distribution. Having domains representing multiple ECM proteins that are individually tunable for concentrations and/or stiffness is already an advance in studying the soft tissue TME of sarcomas such as EwS.

Another challenge in using hydrogels is cell recovery following encapsulation. Several gold-standard techniques assessing biological fate require retrieval of single-cell suspensions or spheroids for flow cytometry, cell sorting, or even gene/protein expression assays. To derive biological meaning, the cells need to be viable, and the cell retrieval technique must not alter their biological state. However, current methods for cell recovery from hydrogels are hampered by the use of harsh agents including Collagenases I, V, XI and Dispase II, along with longer incubation times [63]. Herein, we utilized a biologically invisible enzymatic approach for the digestion of cell-containing hydrogels and the recovery of encapsulated cells thereby maintaining high cell viability for subsequent analyses. Through incorporating an eSrt2A9-sensitive substrate in our crosslinker, we were successfully able to retrieve the encapsulated EwS cells without affecting their viability. Bretherton et al. showed that 2A9 treatment did not alter the transcriptome of mouse cardiac fibroblasts [22]. The lack of change to the transcriptome of cells alludes to the power of utilizing evolved sortases for biomaterial modulation and downstream cell analyses. This unique property in our system allows us to define the differences in the transcriptome of EwS cells as well as to capture their post-transcriptional profile. RNA isolation rendered yields as high as 254.3 ng/μL. Additionally, the RIN being greater than 9 (with 10 being the maximum) for all samples makes it convenient to perform RNA sequencing in the future. While retrieval efficiency was between 15-40% (Fig S3d), the cells obtained are sufficient to perform gene expression and flow cytometry. The eSrt2A9-recognizing motif in our crosslinker is a novel method for EwS cell retrieval. Unsurprisingly, EwS cells cultured in our 3D hydrogels exhibited a more physiologically relevant phenotype compared to 2D cultures, evidenced by trends toward upregulated ECM-related genes (*COL1A1, TNC)* and significantly higher expression of *MMP9* – a known matrix regulator that correlates with a more complex, *in vivo-like* architecture. *MKI67* levels being lower in 3D than 2D is as expected [64]. Fig. 2 illustrates the differences in cell function between 2D and 3D but more importantly, shows that the cells retain viability and that sortase treatment opens avenues for a plethora of other assays.

EwS is conventionally classified as a pediatric bone malignancy. This perception has historically led biomaterial development to predominantly focus on the naturally stiffer bone TME [65,66]. For example, a collagen I- and hyaluronic acid-based highly porous stiff material was exposed to physiologically-relevant mechanical loads to understand biomechanical regulation of EwS cells [67]. Another group utilized 3D printed electrospun polycaprolactone or poly(propylene fumarate) scaffolds to elucidate effects of substrate stiffness, shear stress, and flow perfusion on EwS cells [65,68]. However, this approach fails to model EwS metastases that occur in lungs and other soft tissues [69]. Consequently, ECM changes in the metastatic soft tissue microenvironment have yet to be deeply interrogated in preclinical platforms. Our work brings forth a model that allows researchers to specifically study soft tissue TMEs of metastatic sarcomas with a focus on EwS. The TNC rich microenvironment is particularly relevant for EwS progression, and our work demonstrates the impact of including the TNC-derived peptide AEIDGIEL in the RCT hydrogel design. The region from the FN-III repeat of the TNC protein was chosen due to its overlap with α9β1 integrin engagement, a critical pathway in solid tumors [70]. We observed that the presence of the TNC peptide in RCT hydrogels increased the expression of CD44 and *MMP9* compared to RC hydrogels (Fig. 4). An increase in MMP9 driven by TNC protein has been previously reported in pancreatic cancer [71]. Higher *CD44* and *MMP9* expression were also noted in 3D than 2D (Fig. 2). Evidence in literature showed that an increase in MMP9 led to higher migratory abilities in EwS cells [72]. Furthermore, the CD44-MMP9 complex is known to promote tumor invasion and metastasis [73]. Additionally, high CD44 expression is associated with a more migratory and invasive phenotype in EwS cells [49]. A significant upregulation was also noted in RCT gels for *MMP2* and *LGMN*, genes that together promote cell migration and matrix remodeling [74,75]. This collective increase of metastasis-associated markers in EwS cells within RCT hydrogels suggests that the TNC peptide activates cell state changes in EwS cells, changes that are associated with mesenchymal phenotypes.

The increase in TNC expression by EwS cells in RCT gels further necessitated the need to probe the specificity of the TNC peptide and test whether it can indeed be a surrogate for the whole TNC protein. Mechanistically, our results support the established feed-forward loop between TNC and Src signaling wherein microenvironmental TNC activates Src thereby promoting secretion of TNC by EwS cells [54]. The TNC peptide in the RCT hydrogel reproducibly activated pSRC in two distinct cell lines and this activation was reversed by treatment with the potent Src inhibitor dasatinib. Given that the 10 μM dose of dasatinib may have additional off-target effects, future work will be needed to investigate whether other signaling programs are also activated by the TNC peptide. Nevertheless, blocking the peptide’s primary receptor α9β1 abrogated TNC production by EwS cells thus revealing the key contribution of TNC:integrin binding to the ECM producing tumor cell state. Depletion of α9 has been shown to suppress TNC deposition in rheumatoid arthritis [76]. The ability of the TNC peptide to at least partially recapitulate the effect of the whole TNC protein provides the distinct advantage of precision and tunability over using whole proteins in a synthetic ECM. Employing other integrin-specific blocking antibodies will aid in definitively identifying the receptors mediating the observed effects.

Finally, while the stiffness of our current hydrogels is comparable to soft tissue like the lung, our platform’s tunability allows for independent regulation of mechanics. Future studies can investigate how matrix stiffening – a known driver of malignancy in breast cancer and other solid tumors – interplays with biochemical signals in EwS metastasis. Additionally, encapsulations of single cell suspensions formed large clusters over time representing disseminated tumor cells in a soft tissue niche. Future work may encapsulate spheroids rather than single cells to better model small metastatic colonies. The “plug and play” approach may also be beneficial to understanding the influence of peptides derived from alternative regions of the TNC protein. Gaining a better understanding of the biologic drivers of metastasis is necessary for the development of more specific and effective therapeutic targets. While this work focuses on EwS, the hydrogel system can be used for all ECM-rich soft tissue sarcomas.

## 5. Conclusions

The ECM of solid tumors plays a critical role in governing cancer cell state and metastatic potential. Developing therapeutics to prevent or eliminate metastatic progression requires a more complete understanding of ECM:tumor crosstalk. To that end, we have developed a highly tunable biomimicking 3D hydrogel platform to understand how ECM influences EwS cell behavior. Designed specifically to study the role of TNC in EwS, we created a hydrogel that mimics a TNC-poor ECM using FN- and Col-derived peptides. To simulate a lung metastatic niche, we modified this system to contain a TNC-derived peptide. Additionally, for efficient biologically invisible cell retrieval from the hydrogels, we have incorporated a sortase-degradable crosslinker. Our findings demonstrate that EwS cells require integrin engagement to survive and grow in 3D matrices. More importantly, we observe that the addition of the TNC peptide alters phenotypic properties of encapsulated tumor cells. While our hydrogels were designed to study EwS, the design is highly adaptable to other sarcomas as well as carcinomas that metastasize to lung. In sum, these 3D hydrogels serve as a reductionist mimic of soft-tissue TMEs and enable *ex vivo* studies of the biology of tumor cells in biochemically diverse ECM milieus.

## Supplementary Material

1

Supplementary material associated with this article can be found, in the online version, at doi:10.1016/j.actbio.2026.07.054.

## Figures and Tables

**Table 1:** Sequences, molecular weights, and final working concentrations of azide-modified peptides used in hydrogel formulations.

Sequence	Molecular Weight (g/mol)	Final Concentration (mM)
N_3_-G**RGDS**-NH_2_	600.6	0.64
N_3_-G**DGEA**-NH_2_	557.53	0.12
N_3_-G**GFOGER**-NH_2_	845.92	0.12
N_3_-G**AEIDGIEL**-NH_2_	1026.12	0.44
N_3_-RGPQGIWGQ**LAETG**GRK(N_3_)-NH_2_	2034.06	6

## Data Availability

Data will be made available upon request.
