# Supplementary material for: Modeling Tenascin-C-rich metastatic tumor niches in engineered hydrogel biomaterials
Source: Acta Biomater. Author manuscript; Available in PMC 2026 Sep 1. (PMC13528202; doi:10.1016/j.actbio.2026.07.054)
Supplement: Supplementary file 1 [file NIHMS2202785-supplement-1.pdf]

Supplementary Information

Modeling Tenascin-C-rich Metastatic Tumor Niches in Engineered Hydrogel Biomaterials

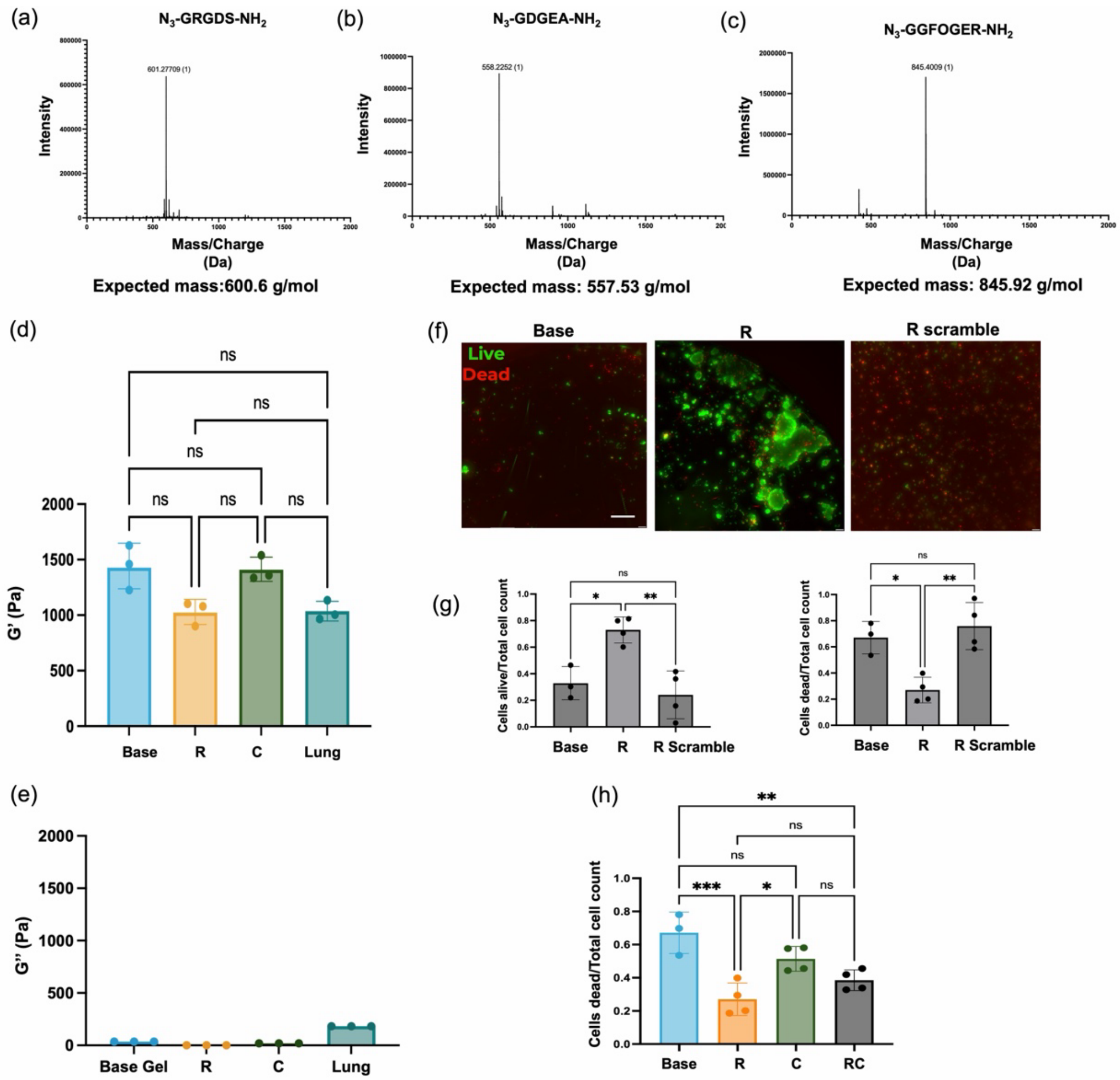

Fig **S1**: Electrospray Ionization Liquid Chromatography Mass Spectrometry (ESI-MS) of azide-modified ECM peptides: (**a**) N_3-GRGDS-NH_2, (**b**) N_3-GDGEA-NH_2, (**c**) N_3-GGFOGER-NH_2. Expected masses were the dominant peaks observed. The charge number of ions is indicated with parentheses. (**d**) No significant differences between storage modulus (G') of base, R, C, and lung observed. Total peptide concentration was kept the same by adding scrambled versions of the bioactive peptide sequences. (**e**) Loss modulus (G") of base gel is approximately 35Pa, R is 0.005Pa, and C is 19Pa. (**f**) Through live/dead assay, EwS cells only survive with RGDS mediated adhesion and die without R or even with the scrambled RGDS peptide. Scale bar=100µm. (**g**) Quantification of live/dead staining demonstrates highest ratio of cells alive with the R gels. The R gels also induce least cell death. (**h**) Quantification of total number of dead cells over total cell count for base, R, C, and RC hydrogel groups. One-way ANOVA, Tukey's post-hoc, n=3 or 4, $*p<0.05$, $**p<0.01$, $***p<0.001$.

(a)

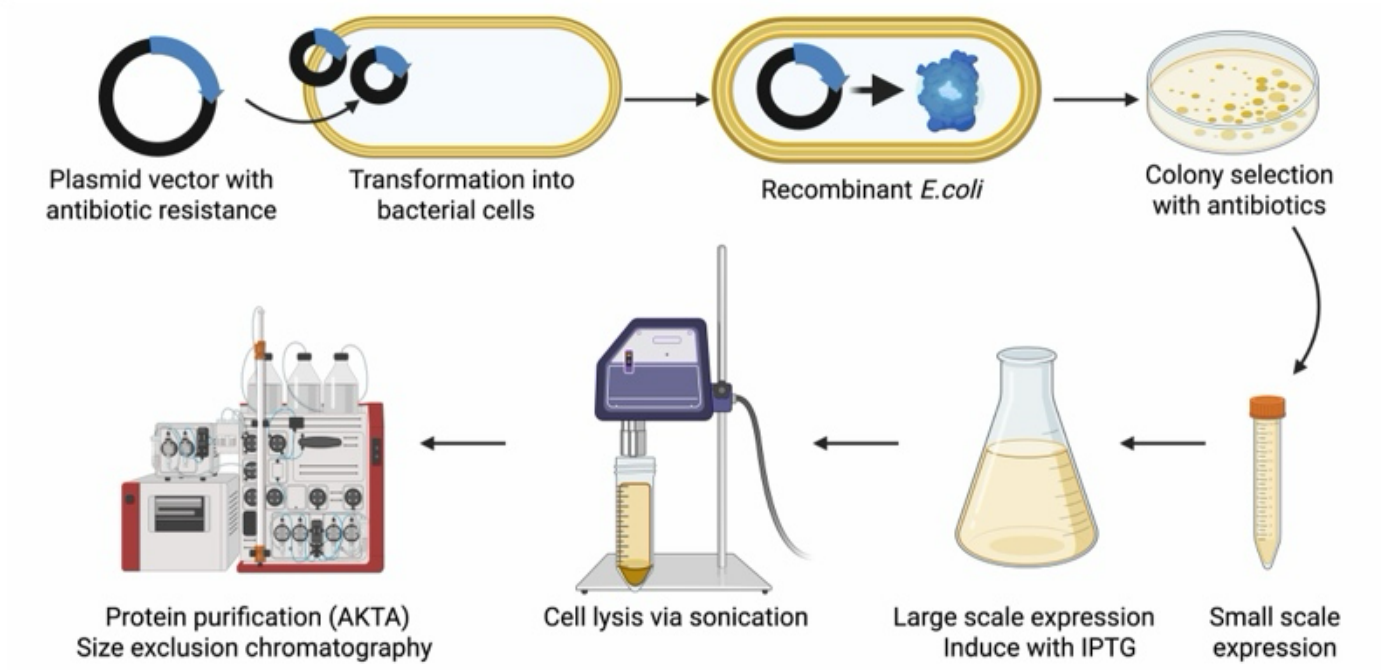

(b)

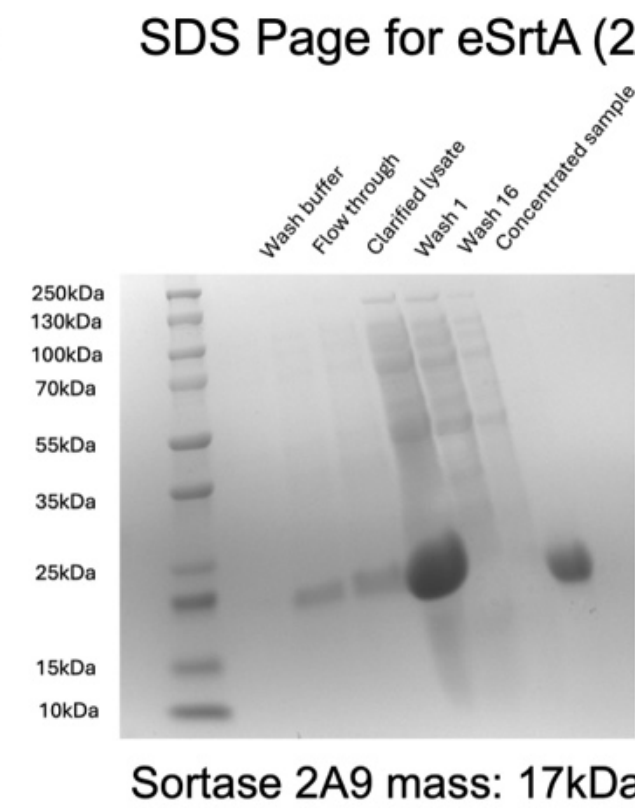

(c)

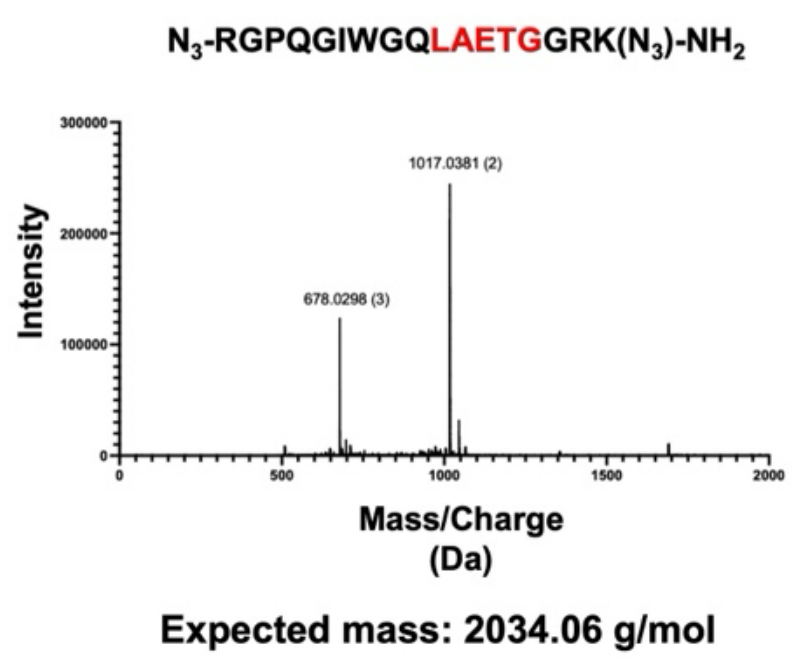

(d)

Sample (2 20µL gels)	RNA concentration (ng/µL)	RNA purity (260/280)	RNA Integrity Number (RIN)
1	174.2	2.02	9.6
2	254.3	2.04	10
3	214.8	2.01	9.8
4	193.9	2.00	9.5

Fig **S2**: (**a**) Schematic for Sortase 2A9 expression and purification. (**b**) Expected mass of the 2A9 sortase protein was observed by SDS-PAGE around 17kDa. (**c**) ESI-MS analysis of the diazide modified peptide with a sortase substrate sequence LAETG. Expected mass was observed as the dominant peak. The charge number of ions is indicated with parentheses. (**d**) RNA yields measured after sortase degradation of hydrogels demonstrate high yield and good purity. RIN was greater than 9 for all samples.

2A9 Expression and Purification

Electrically competent BL21 cells were transfected with eSrtA2A9 plasmids and selected on agar plates containing kanamycin. Luria Broth (LB) (5mL) with kanamycin (50µg/mL) was inoculated with the selected plasmid-containing colony (small scale expression). LB broth with plasmids were allowed to grow overnight at 37°C shaking at 200 rpm. The next day, all 5mL of the plasmid and LB broth was transferred into a 1000mL solution containing LB broth as well as kanamycin (large scale expression). The large volume was allowed to incubate at 37°C shaking at 200 rpm with quantifying optical density (O.D.) values every 30 minutes. Once O.D. reached 0.6, we added isopropyl β-d-1-thiogalactopyranoside (IPTG) (0.5mM) to the culture for inducing protein expression. Following the addition of IPTG the large culture was moved to 18°C, 200rpm for overnight incubation. The next day, cells were centrifuged (4000g, 20mins) to obtain a pellet. The pellet was resuspended in lysis buffer containing 20 mM Tris, 50 mM NaCl, and 10 mM imidazole (pH=7.5) with 1mM phenylmethylsulfonyl (PMSF; TCI, Portland, OR) protease inhibitor. Lysis occurred via sonication (6x, 3min cycle, 30% amplitude; Fisher Scientific

Waltham, MA). The lysate was then clarified by centrifugation for 20mins at 7500g and further filtered with a 0.45um syringe filter (Sartorius, Göttingen, DE). For purification of the clarified lysate, we utilized an ÄKTA Pure 25L FPLC (Cytiva; Marlborough, MA) equipped with a 5 mL HisTrap HP column at a flow rate of 5 mL/min. Equilibration of the column occurred by 5 column volumes of lysis buffer, followed by loading the sample and washing with 8 column volumes of endotoxin removal buffer (20 mM Tris, 50 mM NaCl, 20 mM imidazole, 0.1% Triton-X 114; pH = 7.5) as well as 8 column volumes of wash buffer (20 mM Tris, 50 mM NaCl, 20 mM imidazole; pH = 7.5). His-tagged protein was eluted into a 96 well plate over an 8-column volume gradient of imidazole (20 mM Tris, 50 mM NaCl, 5-250 mM imidazole; pH = 7.5) into 50mL tubes. Protein-containing fractions were dialyzed into PBS over 48hrs. Purified sortase was spin concentrated with an Amicon Ultra 15 centrifugation filter (10,000 Da MWCO) and diluted with 20% glycerol made with PBS to a final concentration of 100uM (2X). Sortase

purity was evaluated by sodium-dodecyl sulfate-polyacrylamide gel electrophoresis (SDS-PAGE).

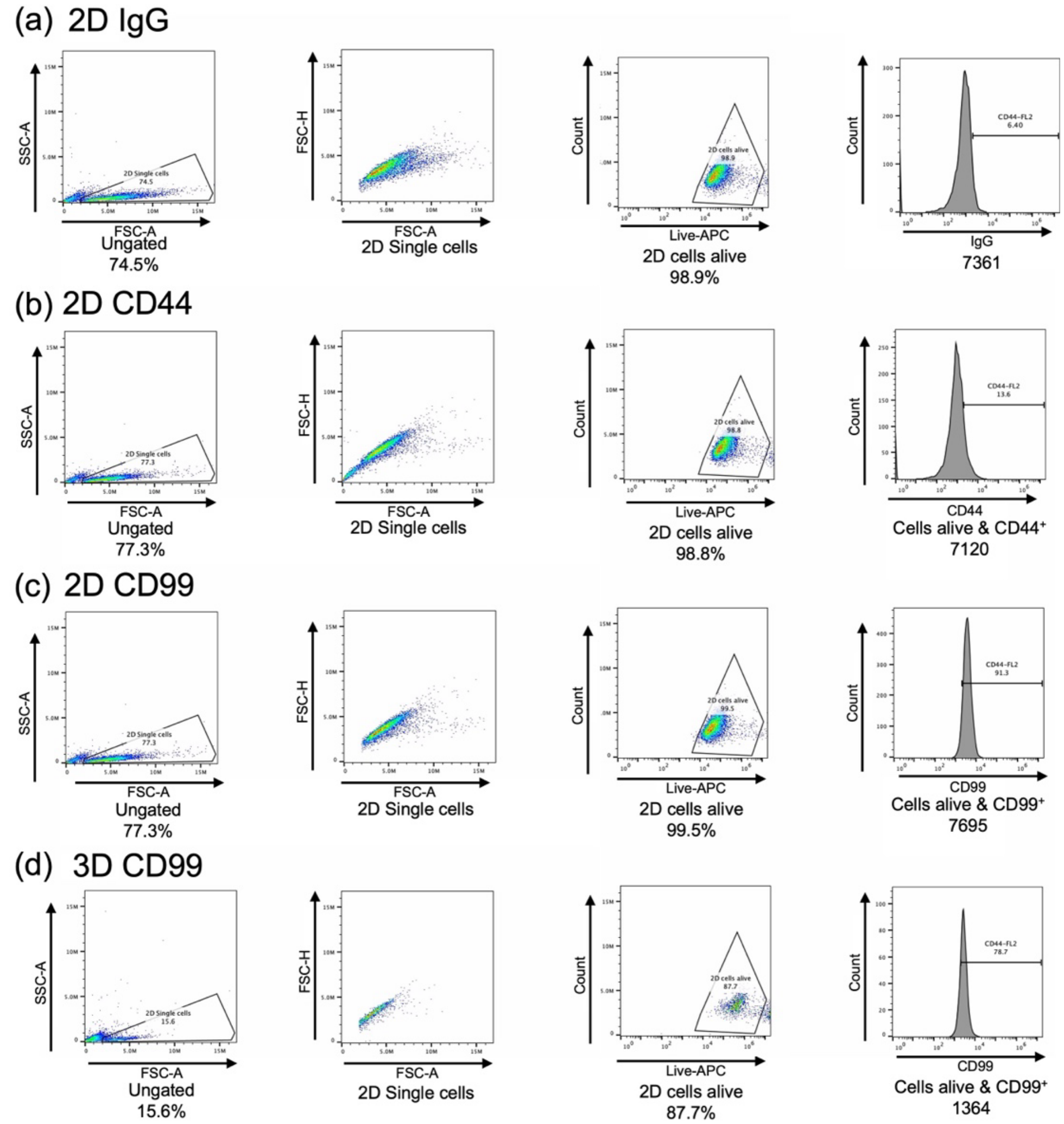

Fig **S3**: Gating strategy for CD44 and CD99 expression comparing 2D vs 3D. After drawing gates for cells, live cells, we created FSC-A vs FSC-H plots to visualize singlets and doublets. Since all cells were single, no gates were drawn for singlets. Gates were then drawn around live cells before quantifying marker expression.

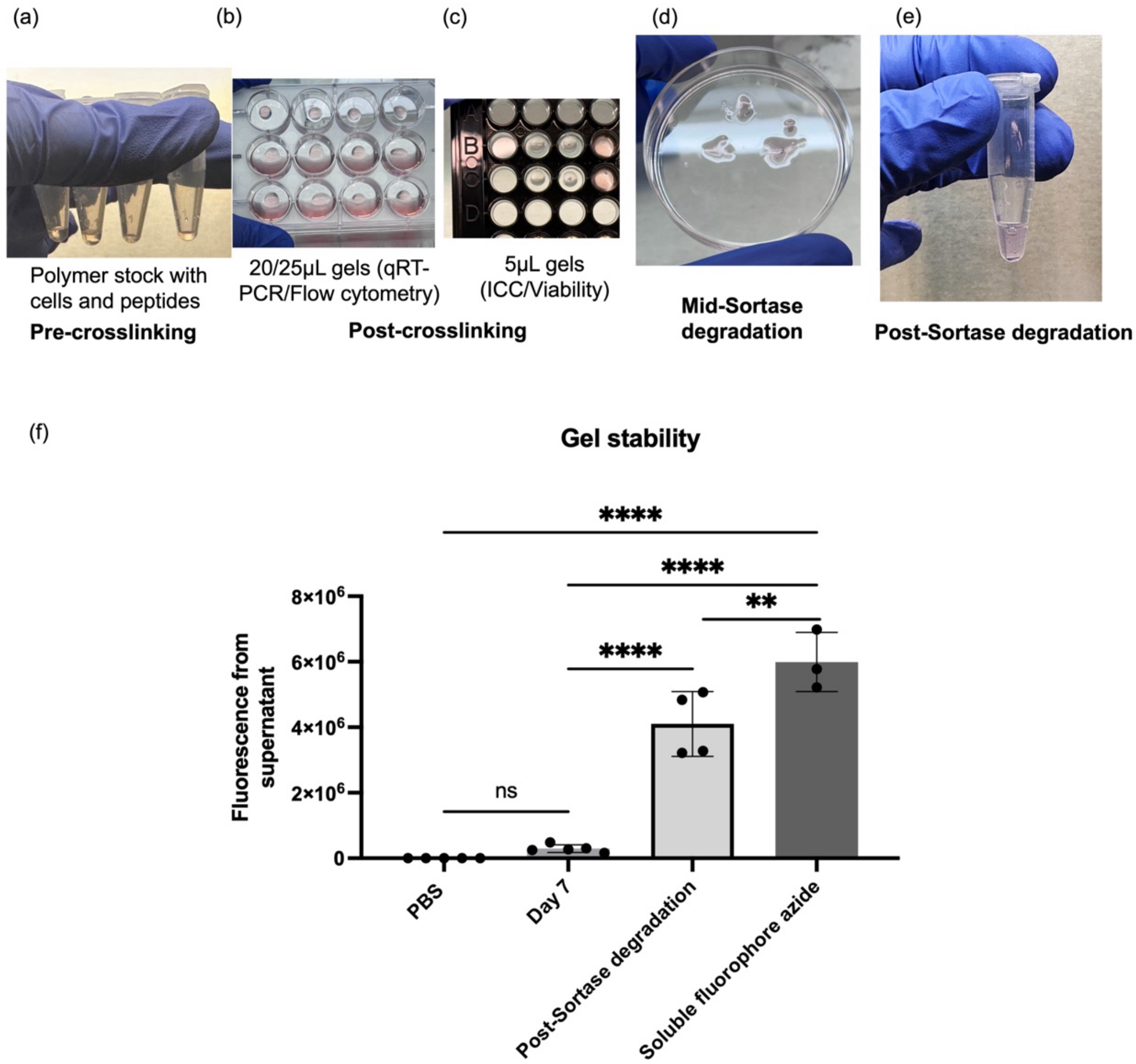

Fig **S4**: Hydrogel crosslinking and gel stability measurements. (**a**) Polymer, peptides, and cells in solution before crosslinking. Gels were then pipetted as (**b**) 20µL (flow cytometry) or 25µL (qRT-PCR) droplets for downstream applications or (**c**) 5µL droplets for immunocytochemistry and viability assays. (**d**) Hydrogels losing structure during sortase degradation. (**e**) Entirely degraded gel with cells in solution. (**f**) Hydrogels are stable over 7 days as depicted by minimal fluorescence from supernatant compared to degraded hydrogels.

Hydrogel stability measurements

Hydrogel stability was measured by conjugating a fluorescent azide 550-Red Oxazine Azide (MW=339 g/mol) (#15319, Active Motif) at the same concentration as the sum of all peptides in RC/RCT hydrogels (1.32mM). Hydrogels were cast as previously discussed with 1.32mM fluorescent azide as the pendant peptide. Gels were immersed in PBS and incubated at 37°C. Fluorescence from the supernatant of crosslinked gels, degraded gels, as well as the fluorescent azide in solution was measured for a week. Fluorescence was measured on a Spectral Plate Reader at excitation 557nm and emission 621nm. While the crosslinked gels had minimal

fluorescence over 7 days, significantly higher fluorescence was detected from sortase degraded gels and by adding the fluorescent azide in solution.

Mesh size calculations

Mesh size of the hydrogels was calculated using the storage moduli of swollen gels, as shown in the following equation:

$$\varepsilon = \left(\frac{RT}{G' N_A}\right)^{-1/3}$$

ε: Mesh size

R: Gas constant, 8.314 m^3 Pa K^{-1} mol^{-1}

T: Temperature (24°C)

G': Storage modulus (Pa)

NA: Avogadro constant, 6.022 × 10^{23} mol^{-1}

For G' ranging from 899Pa-1000 Pa, mesh size = 16-16.6nm.

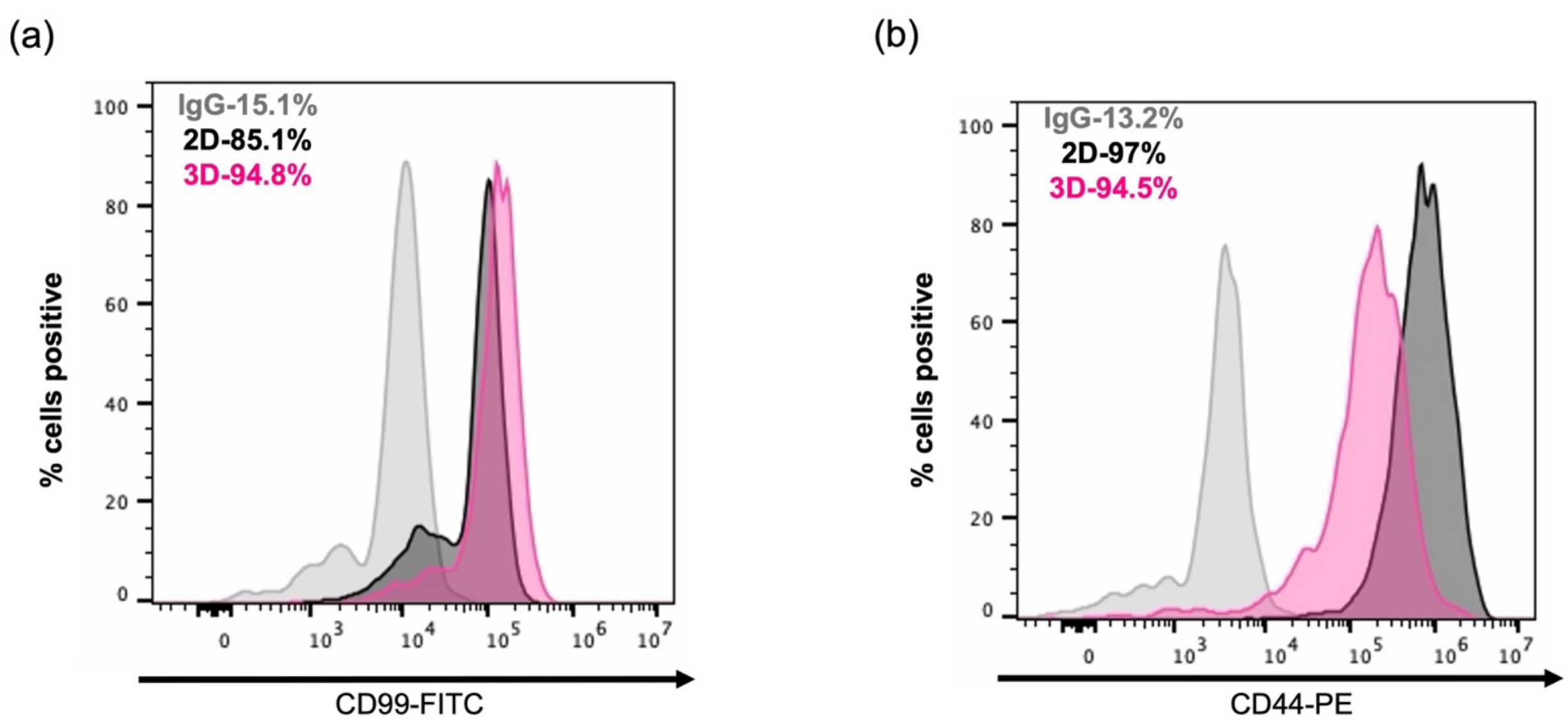

Figure **S5**: CD99 and CD44 expression measured through flow cytometry in PDX305 cells retrieved from 2D TCP or 3D RC hydrogels. (**a**) CD99 expression of PDX305 cells is 85% in 2D and 95% in 3D. Since all EwS cells express CD99, these data demonstrate feasibility of technique. (**b**) There are no differences in CD44 expression by PDX305 in 2D or 3D. Overall CD44 expression is higher in PDX305 cells than EwS cell line CHLA10.

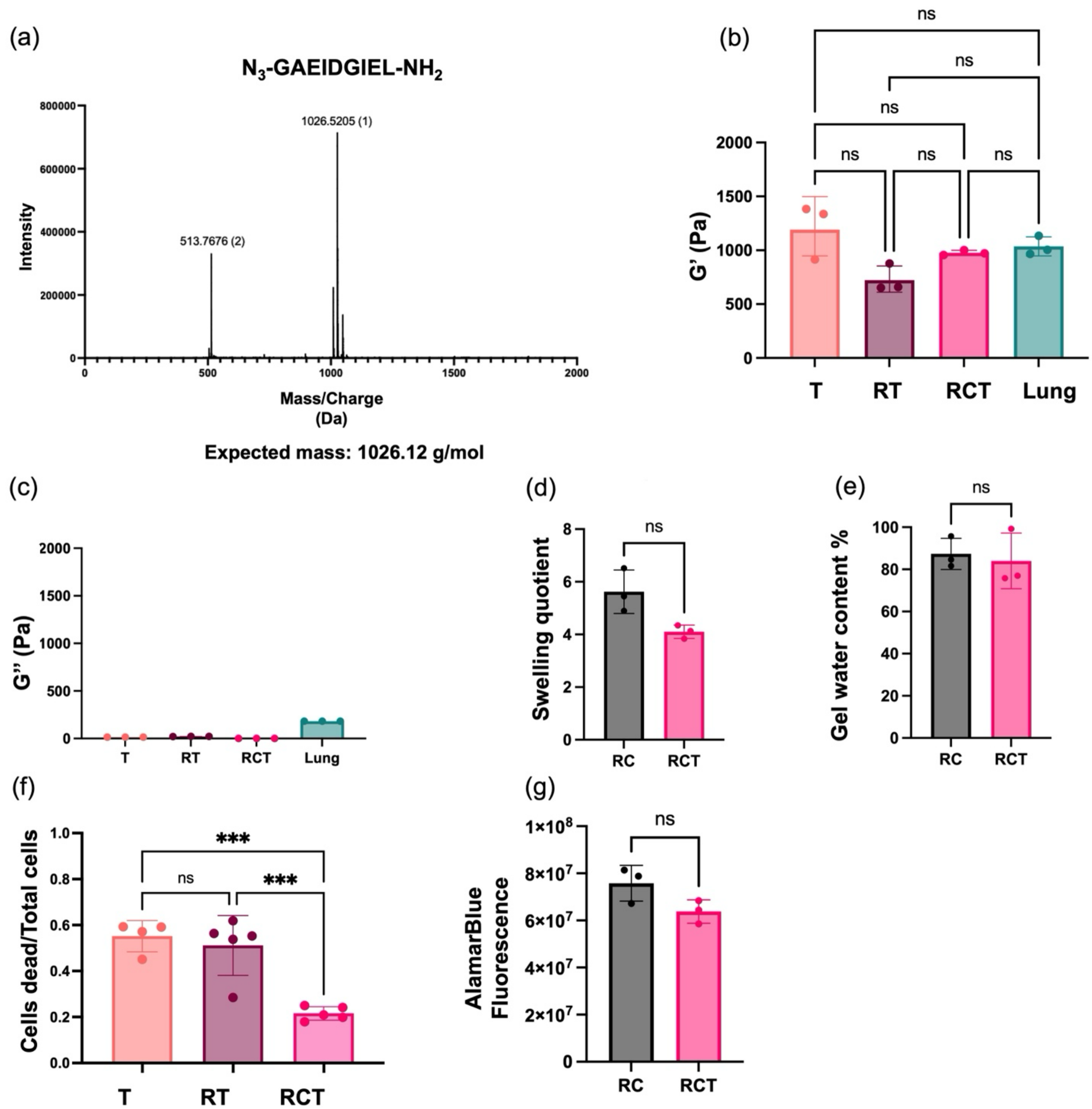

Fig **S6**: (**a**) ESI-MS analysis of the TNC peptide N_3-GAEIDGIEL-NH_2. Expected mass was observed as the dominant peak. (**b**) No significant differences observed in the storage modulus (G') of T, RT, RCT, and lung suggesting no change in stiffness with total peptide concentration being the same. (**c**) Loss modulus (G'') of T, RT, and RCT hydrogels were nearly 0 exhibiting viscoelasticity. (**d**) Swelling ratios between RC and RCT were not significantly different. (**e**) Hydrogel water content calculated from the swollen mass and dried mass were not significantly different between RC and RCT. (**f**) Quantification of dead cells normalized to total cell count demonstrates least cell death in RCT hydrogels. (**g**) AlamarBlue fluorescence levels at end point (day 5) between RC and RCT are not significantly different. These data are previously shown comparing Days 0 to 5 in Figs **1f** for RC, and **3f** for RCT. One-way ANOVA or student's t-tests, Tukey's post-hoc, n=3 or 4, ***$p<0.001$.

Swelling ratio and Water Content

Hydrogels were cast at concentrations of 3mM PEG-BCN:6mM diazide crosslinker between Rain X treated glass slides so they can remain free-floating hydrogels. Gels were allowed to swell in 4mL DI H_20 overnight in petri dishes. Post swelling, excess water was removed by gently dabbing and hydrogel weight was measured. The hydrogels were then transferred to pre-weighed 1.5mL Eppendorf tubes and allowed to dry overnight upon which the dried polymer weight was measured. Gel swelling ratio (Q) and water content were calculated as follows:

$$\boldsymbol{Q} = \frac{M_{gel}}{M_{polymer}}$$

$$Water\ content\ (\%) = 100\% \times \frac{M_{gel} - M_{polymer}}{M_{gel}}$$

Where , M_{gel} = Mass of swollen gel

$M_{polymer}$ = Mass of dried gel

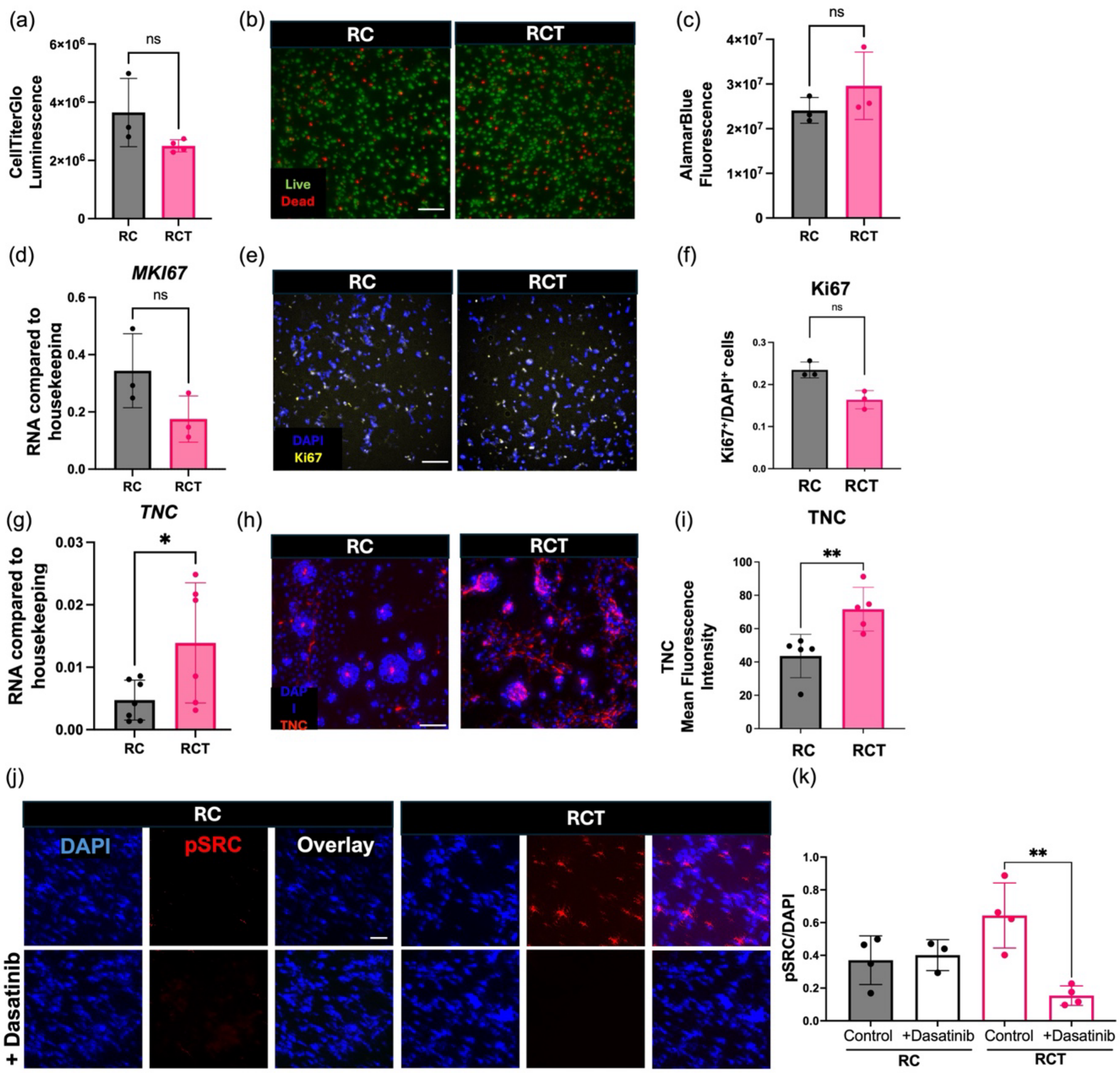

Fig **S7**: PDX305 viability and proliferation are not different between RC and RCT. (**a**) Viability through (**a**) CellTiter-Glo, (**b**) Live/Dead staining, and (**c**) AlamarBlue metabolic measurements are not significantly different between RC and RCT at day 5. Proliferation of PDX305 cells is not different between RC and RCT quantified by (**d**) qRT-PCR of *MKI67* and (**e**) immunocytochemistry of Ki67$^+$ cells. Representative images shown. (**f**) Ki67$^+$ cells were normalized to total DAPI count. Scale bar =100µm; n=3/4; Student's t-tests, *p<0.05. PDX305 upregulates TNC expression through (**g**) qRT-PCR and (**h**) immunocytochemistry in RCT compared to RC. (**i**) Quantification of TNC by mean fluorescence intensity (MFI) is significantly higher in RCT than in RC. Scale bar = 100µm. Student's t-tests; n=6; *p<0.05; **p<0.01. RCT hydrogels upregulate pSRC expression in PDX305 cells and that is blunted with 10µM dasatinib. (**j**) Representative images of RC and RCT with and without dasatinib treatment. DAPI (blue), pSRC (red), and overlay from left to right. Scale bar =100µm. Images are a maximum intensity projection of a 350µm Z-stack. (**k**) Quantification of pSRC over DAPI clusters. One-way ANOVA, tukey's post-hoc, n=3 or 4, **p<0.01.
